# The histone variant H2A.Z is an important regulator of enhancer activity

**DOI:** 10.1093/nar/gkv825

**Published:** 2015-08-28

**Authors:** Mylène Brunelle, Alexei Nordell Markovits, Sébastien Rodrigue, Mathieu Lupien, Pierre-Étienne Jacques, Nicolas Gévry

**Affiliations:** 1 Département de biologie, Faculté des sciences, Université de Sherbrooke, 2500 boulevard de l'Université, J1K 2R1, Sherbrooke, Québec, Canada; 2 Département d'informatique, Faculté des sciences, Université de Sherbrooke, 2500 boulevard de l'Université, J1K 2R1, Sherbrooke, Québec, Canada; 3 Ontario Cancer Institute, Princess Margaret Cancer Centre/University Health Network and Department of Medical Biophysics, University of Toronto, Toronto, Ontario, M5G 1L7, Canada; 4 Centre de recherche du Centre hospitalier universitaire de Sherbrooke, 12e Avenue Nord, Sherbrooke, Québec, J1H 5N4, Canada

## Abstract

Gene regulatory programs in different cell types are largely defined through cell-specific enhancers activity. The histone variant H2A.Z has been shown to play important roles in transcription mainly by controlling proximal promoters, but its effect on enhancer functions remains unclear. Here, we demonstrate by genome-wide approaches that H2A.Z is present at a subset of active enhancers bound by the estrogen receptor alpha (ERα). We also determine that H2A.Z does not influence the local nucleosome positioning around ERα enhancers using ChIP sequencing at nucleosomal resolution and unsupervised pattern discovery. We further highlight that H2A.Z-enriched enhancers are associated with chromatin accessibility, H3K122ac enrichment and hypomethylated DNA. Moreover, upon estrogen stimulation, the enhancers occupied by H2A.Z produce enhancer RNAs (eRNAs), and recruit RNA polymerase II as well as RAD21, a member of the cohesin complex involved in chromatin interactions between enhancers and promoters. Importantly, their recruitment and eRNAs production are abolished by H2A.Z depletion, thereby revealing a novel functional link between H2A.Z occupancy and enhancer activity. Taken together, our findings suggest that H2A.Z acts as an important player for enhancer functions by establishing and maintaining a chromatin environment required for RNA polymerase II recruitment, eRNAs transcription and enhancer-promoters interactions, all essential attributes of enhancer activity.

## INTRODUCTION

Enhancers play a central role in achieving cell-type and cell-state-specific transcriptional programs, by integrating signals (e.g., from developmental, differentiation or hormonal stimuli) through the recruitment of specific transcription factors (TFs) and co-activator complexes in order to coordinate gene regulation of different subsets of target genes (1–3). Enhancers identification is thus crucial to understand physiological and pathological processes, such as the extensive deregulation of gene expression patterns observed in cancer (4). It is now achievable by exploiting the dichotomy of lysine 4 methylation (me) status of histone H3 (H3K4) between enhancers and promoters. Indeed, enhancers are preferentially marked by mono- or di-me of H3K4 (H3K4me1/me2) whereas promoters are marked by di- or tri-me (H3K4me2/me3) (5). In addition, the combined presence of these marks with lysine 27 acetylation of histone H3 (H3K27ac) reveals their active state (2,6–9). Enhancers are also frequently bound by the transcriptional coactivator p300 (2,5,10). The next step to understand the role of this main regulator of gene expression is to elucidate the mechanisms responsible for enhancer functions at the chromatin level.

Chromatin structure and composition regulates gene expression by dynamically limiting the accessibility to DNA, which governs the fine tuning of TF–DNA interactions. Gaining access to DNA wrapped around nucleosome requires DNA unwrapping or nucleosome eviction or sliding. These modifications in chromatin structure can be detected by DNA hypersensitivity to deoxyribonuclease I (DNase I) that degrades accessible DNA more easily than nucleosomal DNA (11). Although the vast majority of sites occupied by TFs occur within accessible chromatin defined by DNase I hypersensitivity (DHS) (12), DHS could be associated with high nucleosome occupancy (13,14), with high turnover of histones (15) and are occupied by unstable nucleosomes containing histone variants H3.3 and H2A.Z (16–18). These results implicitly suggest that rather than nucleosome-free regions, TF-binding sites (TF-BS) are the scene of a continuous process of nucleosomal disruption, essential to maintain their accessibility. Furthermore, the classical view of nucleosome topology in relation to TFs at enhancers, e.g., the two well-defined nucleosomes on each side of the TF-BS, was recently questioned by Kundaje *et al*. (19), who reported a heterogeneity and asymmetry in the deposition of histone modifications and in nucleosome occupancy around TF-BS. To add further complexity, it has been shown that transcriptional activation is correlated with nucleosomal reorganization at enhancers (20–22), although this observation could not be generalized to all TF-bound enhancers tested (23,24). These results suggest that further work is required to understand the role played by chromatin structure and composition in enhancer functions.

Genome-scale studies have reported H2A.Z as a chromatin component at DHS (6,17,25). It is possible that H2A.Z itself could be important for modulating functionalities of enhancers rather than simply marking DHS. For instance, H2A.Z incorporation into chromatin is independent of DNA replication and implies the action of ATP-dependent nucleosome remodelers, namely SRCAP and p400 complexes (26,27). In addition, H2A.Z nucleosomes show a higher susceptibility to asymmetric internal nuclease cleavage *in vivo* and thus protect less DNA than H2A nucleosomes (28). Finally H2A.Z/H3.3 nucleosomes are characterized by hypersensitivity to salt-dependent dissociation that measures nucleosome structure stability (16–18). Since they are localized over regulatory elements, it is tempting to speculate that their instability contributes to chromatin accessibility for TFs binding. Indeed, H2A.Z depletion in murine embryonic stem cells increases overall nucleosome level at p300-intergenic sites and reduces the accessibility of ∼20% of DHS, suggesting a role of H2A.Z in maintaining chromatin accessibility (29). However, the presence of H2A.Z was not directly verified at sites affected by H2A.Z depletion, preventing conclusions about a direct control by H2A.Z on accessibility.

Studies of the androgen receptor (AR), a ligand-activated TF belonging to the nuclear receptors superfamily (NRs), has suggested an implication of H2A.Z in enhancer functions via chromatin reorganization (21). Upon activation, the binding of AR at enhancers enriched for H3K4me2 was found to be associated with a depletion of a central nucleosome present at AR-BS, flanked by a pair of nucleosomes (called the symmetric or bimodal pattern). Importantly, they observed an H2A.Z enrichment at this central nucleosome for five loci tested by ChIP-qPCR. However, the temporal dynamics or persistence of H2A.Z occupancy have not been established neither their functional implications. This is particularly relevant considering the intrinsic properties of H2A.Z nucleosomes, the fact that most NR binding events occur at pre-existing enhancers exhibiting accessible chromatin in unactivated and activated states (24,30–32) and that gene-specific approaches provide examples of both kind of H2A.Z behavior following activation (33–35). Moreover, this symmetric dynamic pattern in nucleosome occupancy has not been observed at estrogen receptor alpha (ERα, a ligand-activated NR) binding sites (ERα-BS) following activation, although DHS has been observed, as if ERα would bind to nucleosomal DNA (24). H2A.Z occupancy and persistence could explain this phenomenon, however they have not been investigated.

In previous work, we observed that H2A.Z is essential for the proper expression of several ERα target genes (34). Interestingly, using ChIP-chip of ERα and H2A.Z on chromosome 17, we identified that ∼16% of ERα-BS are enriched by H2A.Z. Importantly, at the subset of distal ERα-BS enriched for H2A.Z (considered as putative enhancers), H2A.Z is present prior activation and its overall level tends to remain constant after activation, emphasizing a potential role of H2A.Z in shaping the local chromatin environment at enhancers in unactivated and activated states. Similarly, H2A.Z level remains constant at a constitutive DHS bound by the glucocorticoid receptor (GR, a ligand-activated NR) following activation (33). In contrast, the level of H2A.Z following activation is slightly reduced at inducible DHS bound by GR (33) or at few selected ERα enhancers whose chromatin environment depends on the pioneer factor FOXA1 (34–36), whereas its level is increased at one proximal ERα-BS (34). These results suggest different mechanisms that could depend on the chromatin landscape and on the localization of H2A.Z and/or ERα-BS. A substantial challenge in the identification of H2A.Z-specific functions at enhancers is thus imposed by the fact that H2A.Z is found at promoters and enhancers, both harboring distinct epigenetic landscapes (2,5–6); and that there are discrepancies in the literature regarding whether or not H3K4me3 could be found at enhancers (3,5–6,25,29,37). The chromatin composition and/or the localization of the TF-BS relative to Transcription Start Site (TSS) could therefore influence and confuse the interpretation of the specific role of H2A.Z at enhancers.

Thus, in spite of these significant studies, our current understanding of the role of H2A.Z at enhancers is still rudimentary. In this study, we aimed to characterize at a genomic scale the unexplored aspects of the relationship between H2A.Z and enhancer marks, and the influence of H2A.Z on local chromatin environment and structure at inducible enhancers. We used the estrogen signaling pathway of MCF-7 cells, a breast cancer model, which allowed us to study the chromatin and transcriptional mechanisms in a dynamic context. In these cells, a transient stimulation by 17β-estradiol (E2) induces ERα binding, predominantly at distal enhancers, and consequently a specific transcriptional program (38). More specifically, we performed chromatin immunoprecipitation of H2A.Z, H3K4me1, H3K27ac and H3K4me3 at nucleosomal resolution followed by deep sequencing (MNase ChIP-seq) to determine the chromatin composition at ERα-BS. Moreover, using an unsupervised learning method (19) to analyze patterns and potential dynamic transitions in chromatin composition and structure, we examined the influence of H2A.Z and transcriptional activation on the local nucleosome positioning at enhancers. We also scrutinized various existing genomic datasets to determine the influence of H2A.Z on the chromatin environment at enhancers. Finally, we performed ChIP-qPCR and RT-qPCR at selected enhancers following H2A.Z depletion to address specific mechanisms.

## MATERIALS AND METHODS

A detailed description of the methods is available in the Supplementary Material. Briefly, MCF-7 cells were grown in Dulbecco's modified Eagle's medium (DMEM) (Wisent) supplemented with 10% fetal bovine serum (FBS) and antibiotics. Before experiments, cells were hormone deprived for 3 days in DMEM without phenol red (Wisent) supplemented with 5% charcoal-stripped FBS. Cells were then treated with 100 nM of 17β-estradiol (E2, Sigma) or with vehicle (EtOH) for 30 min. ChIP assays were performed as described previously (39), except for chromatin preparation that was adapted to allow MNase digestion. Sequencing libraries were prepared according to the Illumina library preparation protocol except for size selection of DNA fragments and for DNA recovery that were achieved as described previously (40). Alignment of sequencing reads onto the human genome (Build 36.1, hg18) was performed using BWA (Burrows–Wheeler Aligner) (41). Two biological replicates for each sample were generated and combined for subsequent analyses considering that their Pearson correlation coefficients were above 0.9 (Supplementary Figure S1). Wiggler tool (42) was used to uniformly process and normalize MNase ChIP-seq data. Significantly enriched regions were detected using MACS (43). All the public datasets used in this study and their processing steps are described in Supplementary Material. Distal and proximal regions to TSS were defined respectively as more or less than 3 kb of known TSS (a list of unique TSS from the combination of RefSeq and UCSC genes). The K-means clustering of ChIP-seq read density centered on TSS-distal nonpromoter ERα summits were performed using seqMINER (44). The unsupervised pattern discovery at ERα-active enhancers were performed according to the shape of associated H3K4me1, H2A.Z or MNase-sequencing signals, using CAGT (Clustered AGgregation Tool) (19).

## RESULTS

### H2A.Z marks ERα-active enhancers associated with E2-regulated genes

Considering that potential unannotated promoters could bias the interpretation of the role of H2A.Z at enhancers when the classification is only guided by gene annotations, and to clarify the relationship between H2A.Z and H3K4me3 in regard to enhancers, we analyzed both proximal and distal H2A.Z-enriched regions, as well as distal ERα-BS (45). We observed that regions co-enriched for H2A.Z and H3K4me3 mostly harbor promoter properties, regardless of whether these regions are proximal or distal to a known TSS (Supplementary Figure S2), indicating that distal regions are likely to be contaminated by unannotated promoters. As a consequence, the regions enriched for H3K4me3 were removed and we focused our analyses on a conservative list of TSS-distal nonpromoter ERα-BS (see Supplementary Material for further details).

**Figure 1.**
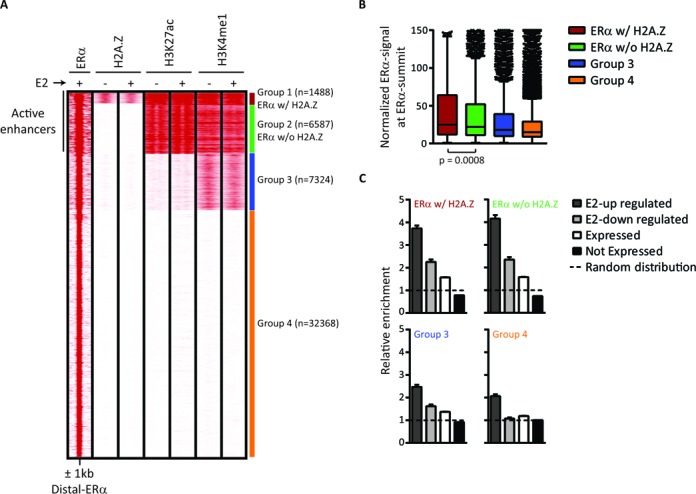
H2A.Z enrichment is restricted to a subgroup of active enhancers. (**A**) K-means clustering of TSS-distal nonpromoter ERα-BS (45), H2A.Z, H3K27ac and H3K4me1 in the absence or presence of E2. Four distinct groups are derived: ERα w/ H2A.Z active enhancers (group 1, red), ERα w/o H2A.Z active enhancers (group 2, green), weak enhancers (group 3, blue) and potential inactive ERα-BS (group 4, orange). (**B**) Distribution of the normalized ERα intensity level for each ERα group. The signal of ERα is significantly higher in ERα w/ H2A.Z (+E2, *P*-value < 0.05, Mann-Whitney test). (**C**) Genes were split in four categories based on expression microarray results before and after E2 stimulation. The ERα-BS were associated to the genes based on their regulatory domain (as in GREAT (64)). The relative enrichment of ERα in each category, compared to a random distribution, is shown for each ERα group.

To determine chromatin composition at these ERα-BS, we performed K-means clustering (44) on ERα, H2A.Z and enhancer marks. Four groups were identified based on the similarity of their chromatin states (Figure 1A and Supplementary Figure S3). H3K4me1 and H3K27ac enrichment suggest that groups 1 and 2 represent active enhancers, group 3 weak enhancers and group 4 putative inactive ERα-BS. Importantly, H2A.Z enrichment occurs exclusively in association with H3K27ac and is restricted to ∼20% of active ERα enhancers (group 1, hereafter referred as ERα w/ H2A.Z), that show significantly higher level of ERα than active enhancers where H2A.Z is not enriched (group 2, hereafter referred as ERα w/o H2A.Z) (Figure 1B). Moreover, ERα w/ and w/o H2A.Z are more enriched than groups 3 and 4 on E2-regulated genes territories (Figure 1C and Supplementary Material), supporting their classification as active enhancers. Interestingly, the E2-induced binding of ERα is not accompanied by drastic overall changes in the level of the epigenetic signals analyzed (Figure 1A and Supplementary Figure S3A). Among the 10% of ERα-BS showing the strongest signal, we thus selected five loci from each group of ERα w/ and w/o H2A.Z and examined their temporal enrichment of H2A.Z by ChIP-qPCR. Supplementary Figure S4 shows genome browser snapshots of these ten loci. As expected, we observed a higher level of H2A.Z occupancy at ERα w/ H2A.Z than w/o H2A.Z and did not detect any major fluctuation in H2A.Z level over a 60 min time course (Supplementary Figure S3C).

**Figure 2.**
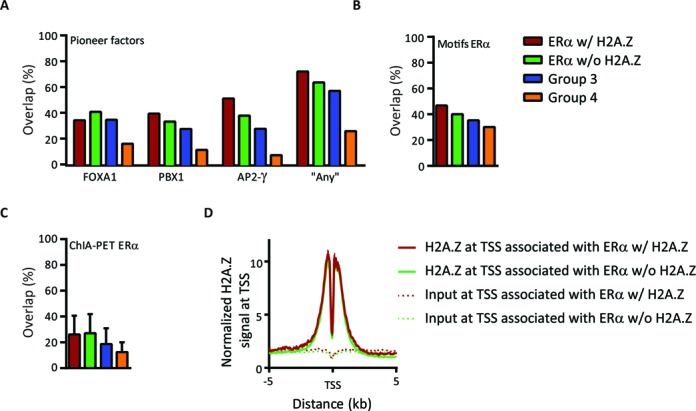
Characterization of ERα groups (**A**–**C**). The overlap proportion between ERα groups and pioneer factors enriched regions (A), the ERE motifs (B), or the ERα engaged in long-range chromatin interactions (C). ‘Any’ represents at least one of the FOXA1, PBX1 or AP2-γ-binding sites (47,48). The mean ± SEM of two replicates of ChIA-PET data of ERα are represented. (**D**) The average profiles of H2A.Z normalized signal over the TSS associated with ERα w/ and w/o H2A.Z.

Pioneer factors are a particular class of TFs that dictate global chromatin structure and facilitate cell-type specific recruitment of other TFs (1). ERα binding at several enhancers requires the presence of pioneer factors such as FOXA1 (46), PBX1 (47) and AP-2γ (48). Considering that H2A.Z has been shown to be enriched at FOXA1-BS (49) and that H2A.Z depletion may decrease FOXA1 binding (34), we investigated whether the higher level of ERα in ERα w/ H2A.Z enhancers could be a consequence of being located more often in the vicinity of pioneer factors BS. We observed that ∼40% of ERα w/ and w/o H2A.Z overlap with FOXA1-BS, and ∼70% overlap with at least one of the pioneer factors tested (47,48) (Figure 2A), eliminating that possibility. Also, these observations discard the hypothesis that pioneer factor's effects on chromatin landscape could explain why only some of the ERα-BS are occupied by H2A.Z. In addition, more than ∼40% of both groups overlap with at least one ERE motif (50) (Figure 2B and Supplementary Figure S5A), and enriched motifs were not considerably different between both groups (Supplementary Table 1). These results suggest that the lower level of ERα signal in ERα w/o H2A.Z seems not a consequence of a higher proportion of indirect ER-binding events via a tethering mechanism.

Since H2A.Z has been shown to be preferentially enriched at regulatory regions such as proximal promoters (6,17,25,29,51), we further investigated whether the presence of H2A.Z at a particular group of ERα enhancers could be a consequence of a misleading ChIP enrichment of H2A.Z belonging to other genomic regions that interact with enhancers. We first verified the overlap of our groups with ERα sites engaged in long-range chromatin interactions (52) and found no difference between ERα w/ and w/o H2A.Z (Figure 2C). Moreover, H2A.Z signal level at TSS associated with these ERα is also similar (Figure 2D), eliminating that possibility. Furthermore, the saturation level was mostly reached in H2A.Z ChIP-seq experiments (Supplementary Figure S5B), confirming that the low level or absence of H2A.Z at most of the ERα-BS is not related to sequencing depth. Hence, the level of H2A.Z from an independent dataset recently published by others (53) also supports the ERα w/ H2A.Z group identified here (Supplementary Figure S5C). Thus, ERα definitively binds two kinds of active enhancers, one with H2A.Z and one without H2A.Z. In absence of E2, both have predetermined basal state of H3K4me1 and H3K27ac enhancer marks, as well as H2A.Z (Figure 1A and Supplementary Figure S3). A comparison between the groups of ERα w/ and w/o H2A.Z thus provides an opportunity to explore the specific role of H2A.Z at active enhancers.

### H2A.Z does not influence the local nucleosome positioning at ERα-active enhancers

**Figure 3.**
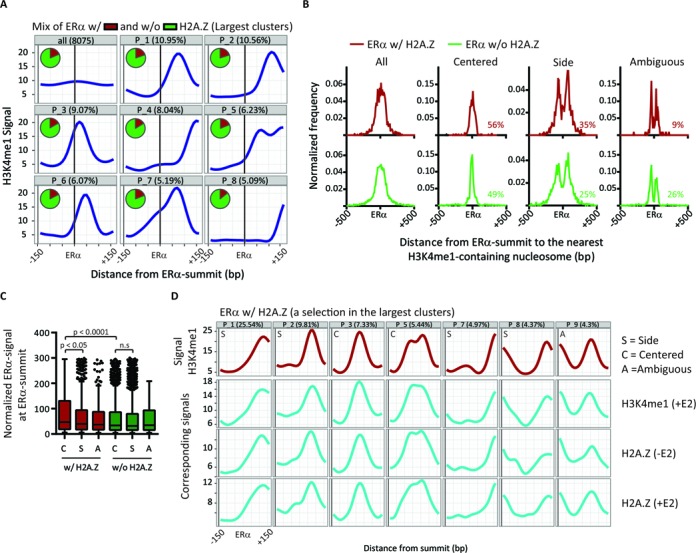
H2A.Z does not introduce a distinct organization of the nucleosomes at ERα-active enhancers. (**A**) Nucleosome position shapes of the largest clusters of the H3K4me1 signal in 300 bp windows centered on ERα summits of ERα-active enhancers. The proportion of ERα w/ (red) and w/o (green) H2A.Z in each cluster is shown in pie charts. (**B**) The normalized frequency of the distance between ERα summit and the nearest nucleosome summit according to their classification as centered, side or ambiguous. The proportion of ERα-BS in each category is shown in the right lower corner of each graph. See Supplementary Figure S6A for typical examples of the classification. (**C**) The normalized ChIP-seq intensity level of ERα at ERα summit (w/ and w/o H2A.Z) according to their classification as centered (‘C’), side (‘S’) or ambiguous (‘A’). The signal of ERα is significantly higher in the centered category of ERα w/ H2A.Z (+E2, p-value of the Mann-Whitney tests are showed directly on the graph). (**D**) Dominant H3K4me1 signal shapes -E2 (red curves) in regions of ERα w/ H2A.Z and the corresponding signals of H3K4me1 +E2 as well as H2A.Z -/+E2 (blue curves). The classification of each pattern is shown in the left upper corner of each graph. The Supplementary Figure S6B contains the 10 largest clusters and the corresponding signals of other histone marks tested.

To determine the influence of H2A.Z on local nucleosome positioning at ERα-active enhancers, we performed unsupervised pattern discovery using CAGT (19). CAGT stratifies the diversity in nucleosomal organization around TF-BS into distinct clusters sharing a similar shape of local nucleosome occupancy, independently of signal level. Moreover, patterns that are mirror images of each other can be oriented in the same direction to distinguish artificial symmetry. We reasoned that if H2A.Z affects nucleosome organization, one or more clusters should be enriched for the ERα w/ H2A.Z group, when performing clustering on all ERα-active enhancers using a common histone mark such as H3K4me1. As previously reported for other TFs (19), CAGT aggregated the H3K4me1 signal around ERα-active enhancers into different clusters of patterns, revealing an heterogeneity and asymmetry in the local nucleosome positioning around ERα summits (Figure 3A). Surprisingly, the proportion of ERα w/ and w/o H2A.Z in each cluster remains constant (∼20 and ∼80%, respectively). No clear relationship is thus observed between H2A.Z presence and a specific shape of nucleosome positioning, suggesting that H2A.Z does not introduce a unique nucleosome organization at ERα-active enhancers.

To confirm this result, H3K4me1 signal was independently clustered on ERα w/ H2A.Z and w/o H2A.Z, and the resulting clusters were classified into three categories: centered, side and ambiguous (Supplementary Material). The computed frequency of the distance between each ERα summit and the nearest positioned nucleosome confirms the classification accuracy (Figure 3B and Supplementary Figure S6A for typical examples). However, ERα w/ and w/o H2A.Z have similar proportion in each category (∼50% of centered and ∼30% of side). Interestingly, the intensity level of ERα is significantly higher at ERα w/ H2A.Z classified as centered than those classified as side, or compared with centered ERα w/o H2A.Z (Figure 3C). This indicates that the centered category w/ H2A.Z is not constituted of low-binding events, and suggests that even if the presence of H2A.Z does not favor a specific configuration of the nucleosomes at ERα enhancers, H2A.Z could introduce a particular chromatin environment or structure.

To validate our observations and confirm that the H2A.Z signal is consistent with the observed patterns, we aligned its signal on clusters generated using the H3K4me1 signal. As shown in Figure 3D, the same typical shapes are observed and H2A.Z signal correlates with H3K4me1 signal. The H3K27ac and the control MNase-seq input signals, as well as the H3K4me2 signal previously published (24) also give similar patterns (Supplementary Figures S6B and S7A). We also directly clustered the H2A.Z signal and detected the same typical shapes as those obtained from H3K4me1 clustering and the H3K4me1 signal is consistent with H2A.Z patterns (Supplementary Figure S7B). Additionally, we conducted a MNase-sequencing experiment, using paired-end sequencing, providing more accurate nucleosome positions, again confirming that histone marks and H2A.Z patterns correlate with nucleosome occupancy (Supplementary Figure S8). However, as ChIP experiments reflect the average nucleosome occupancy in a cell population, it is possible that the chromatin marks may not be on the same nucleosome. Finally, the lack of difference observed in the shapes of chromatin marks between basal and E2-stimulated states suggests no or little displacement of nucleosomes at ERα-active enhancers following ERα-binding (Figure 3D and Supplementary Figures S6B, S7 and S8). Together, these results demonstrate that H2A.Z does not introduce a distinct nucleosomal organization at ERα-active enhancers, and raise the intriguing possibility of understanding why only some of those are occupied by H2A.Z.

### Chromatin accessibility and DNA methylation are close partners for the regulation of chromatin at ERα-active enhancers enriched by H2A.Z

**Figure 4.**
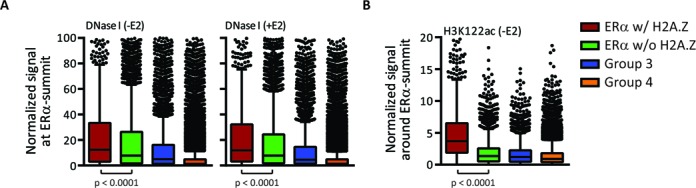
H2A.Z at ERα-active enhancers is associated with chromatin accessibility and instability. (**A** and **B**) Distribution of DNase I normalized signal (A) or H3K122ac ChIP-seq normalized signal (B) at ERα summits of each ERα group. In both cases, the signal is significantly higher in ERα w/ H2A.Z (*P*-value < 0.0001, Mann-Whitney test).

To further explore the role of H2A.Z in chromatin structure at ERα-active enhancers and to get an extensive view of chromatin features that could discriminate H2A.Z-occupied versus -unoccupied ERα-BS, we integrated several existing genomic datasets. Analyses of DNase I sequencing data (12) revealed that ERα w/ H2A.Z show significantly higher accessibility than ERα w/o H2A.Z, for both basal and E2-stimulated states (Figure 4A). Moreover, no major fluctuation is observed in accessibility level following E2 stimulation (Supplementary Figure S9A), in accordance with previous reports (24,30,32). This suggests that one of the effects of H2A.Z on ERα chromatin environment could be to introduce an unstable chromatin structure, as previously demonstrated (16–18).

It has been recently shown that H3K122ac, a modification within the globular domain of H3, is implicated in transcriptional activation, most likely by affecting histone-DNA binding and histone eviction (53). H3K122ac co-occurs with H2A.Z presence at TSS and is also enriched at distal-DHS co-occupied by the transcriptional coactivators p300/CBP, as well as at distal ERα/p300-BS (53). H2A.Z is also enriched in H3K122ac-containing nucleosomes. Hence, it has been proposed that H3K122ac might contribute to the instability of H2A.Z/H3.3-containing nucleosomes (53). Since H3K122ac is well correlated to both H2A.Z and H3K27ac presences (53), we therefore delineated its potential contribution to instability at ERα-active enhancers using a genome-wide dataset previously published (53). In absence of E2 stimulation, H3K122ac is already present at ERα w/ H2A.Z, and its signal level is significantly stronger than the one observed at ERα w/o H2A.Z (Figure 4B and Supplementary Figure S5E), although both ERα w/ and w/o H2A.Z groups have similar overlap with p300- or CBP-enriched regions (54) (Supplementary Figure S9B). These results suggest that ERα w/ H2A.Z regions have a chromatin structure distinct from ERα w/o H2A.Z, in which H2A.Z and H3K122ac could act together on nucleosome dynamics, even prior to E2 stimulation.

**Figure 5.**
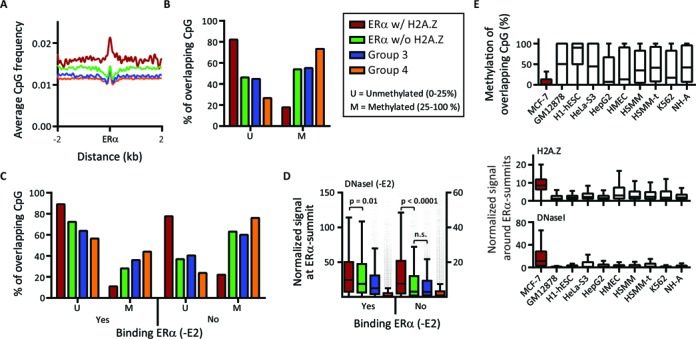
H2A.Z is associated with hypomethylated DNA at active ERα enhancers. (**A**) The smoothed average profiles of CpG dinucleotide occurrences over ERα summits. (**B** and **C**) The proportion of DNA molecules that exhibit cytosine methylation at specific CpG dinucleotide in 600 bp windows centered on ERα summits, for complete ERα groups (B) or for ERα groups split according to their ERα occupancy status by ERα in the absence of E2 (‘yes’= prebound, and ‘no’= not prebound) (C). (**D**) Distribution of the DNase I normalized signal at ERα groups split according to their ERα occupancy status in the absence of E2 (‘yes’= prebound, and ‘no’= not prebound). The signal of DNase I is significantly higher in ERα w/ H2A.Z not prebound by ERα in the absence of E2 (*P*-value < 0.0001, Mann-Whitney test). (**E**) The distribution of DNA methylation status (top panel), normalized signals of H2A.Z (middle panel) and normalized DNase I signal (bottom panel) of different cell lines around ERα w/ H2A.Z genomic regions identified in MCF-7 cells.

DNA methylation status of a genomic region might also affect its chromatin structure. Indeed, an inverse relationship exists between DNA methylation patterns and chromatin accessibility (12,55). Hence, TF binding, including ERα, can be strongly influenced by the methylation status of CpG dinucleotides within their binding sites (56,57). In addition, the distribution of H2A.Z and DNA methylation at TSS and in gene bodies is anticorrelated, H2A.Z protecting DNA from methylation and DNA methylation excluding H2A.Z deposition (58,59). Since hypomethylated DNA is globally observed at enhancers (60–62), we suggest that H2A.Z plays an important role in this process. To test this hypothesis, we first computed the CpG content of ERα-BS and observed that ERα w/ H2A.Z are more frequently located in CpG-enriched regions than other groups (Figure 5A and Supplementary Figure S10A), and not because of a higher overlap with CpG islands (Supplementary Figure S10B). This local increase of CpG density suggests that DNA methylation could be one of the chromatin features contributing to the formation of ERα w/ H2A.Z. We thus analyzed reduced representation bisulphite sequencing data from ENCODE (62), which provides quantitative CpG methylation status at the base pair resolution, expressed as the percentage of methylated cytosine across the cell population. We observed that 82% of CpG found in ERα w/ H2A.Z are un/low-methylated (defined as <25% of cytosine showing methylation), compared with 46% of CpG localized in ERα w/o H2A.Z regions (Figure 5B and Supplementary Figure S10C–E). This result reveals an association between H2A.Z and hypomethylated DNA at active ERα enhancers, and highlights that the overall hypomethylation observed at enhancers might be explained by a mix of unmethylated regions containing H2A.Z and regions where H2A.Z is absent showing much variable methylation level.

It has been suggested that the methylation status of TF-BS is inversely correlated with their occupancy status by TFs (12,61). To test this, ERα-BS were divided according to their occupancy status by ERα in absence of E2. We observed that ∼40% of both ERα w/ and w/o H2A.Z groups show ERα binding in the basal state (Supplementary Figure S10F), but at a much lower level in all four groups than after E2 stimulation (Supplementary Figure S10G). These E2-independent binding of ERα might be partially explained by the presence of growth factors such as epidermal growth factor (EGF), naturally present in the FBS that induce binding of ERα in absence of E2 via phosphorylation of the ERα N-terminal region (63) (Supplementary Figure S10H). As shown in Figure 5C (and Supplementary Figure S10I), the majority of the sites bound by ERα in absence of E2 are poorly methylated in all four groups, suggesting that ERα could maintain this un/low-methylation level as previously proposed for other TFs (12,61). Conversely, only ERα w/ H2A.Z not prebound by ERα are mostly unmethylated, suggesting that H2A.Z could be necessary to maintain the un/low-methylation state of ERα-BS left vacant after a transient E2 stimulation. Importantly, this observation is independent of the localization of ERα-BS relative to gene body (Supplementary Figure S10J), effectively discarding an imbalance in the distribution of ERα-BS in hypermethylated gene bodies. Moreover, ERα w/ H2A.Z not prebound in absence of E2 show a significantly enriched DNase I signal compared with ERα w/o H2A.Z (Figure 5D). This association might point toward a potential implication of H2A.Z to maintain the accessibility of those regions.

We also explored these ERα w/ H2A.Z-associated chromatin features in nine other cell lines, including primary human mammary epithelial cells (HMEC) (3,12,62). Interestingly, none of the cell lines show a methylation status similar to MCF-7 cells, and the H2A.Z enrichment as well as the higher chromatin accessibility are also cell-type specific (Figure 5E). We then focused on ‘high-confidence’ ERα w/ H2A.Z un/low-methylated enhancer not prebound by ERα in absence of E2 (Supplementary Material), and explored their predicted functional targets trying to explain the necessity for the cells to keep those sites ready. Gene ontology analyses (64) revealed that enhancers associated with H2A.Z are enriched on the territories of genes involved in cellular proliferation, adhesion and migration pathways when compared with ERα w/o H2A.Z enhancers (Supplementary Table 2), suggesting that this subset of enhancers could be associated with cancer establishment or progression. Together, these results reveal a novel relationship between H2A.Z, chromatin accessibility, DNA methylation and ERα-binding patterns at enhancers, and suggest that H2A.Z and DNA methylation could be linked to cell-selectivity of chromatin accessibility that models the chromatin landscape to guide the action of inducible TFs. Moreover, DNA methylation might be the epigenetic feature that constrains H2A.Z localization at particular enhancers.

### H2A.Z is required for the recruitment of RNA polymerase II and cohesin complex at active enhancers

**Figure 6.**
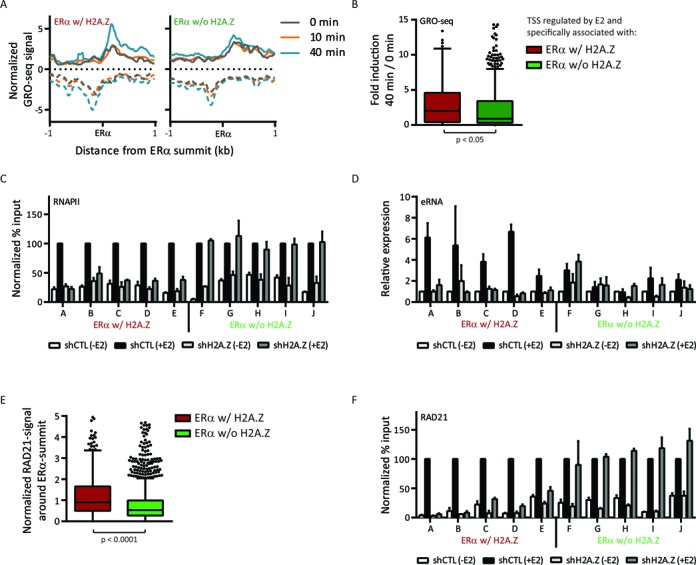
H2A.Z helps the recruitment of RNA polymerase II and cohesin complex as well as transcription at active enhancers. (**A**) Average profiles of strand-specific GRO-seq signal over intergenic ERα-active enhancers at 0 (grey), 10 (orange) and 40 (blue) min after E2 stimulation. (**B**) Distribution of the changes in the expression of E2-regulated genes, specifically associated with ERα w/ or w/o H2A.Z (considered to be their direct E2-regulated target genes, common targets were discarded from this analysis), following a 40 min of E2 stimulation. The transcription of the E2-regulated genes specifically associated with ERα w/ H2A.Z is more induced following E2 stimulation (*P*-value < 0.05, Mann-Whitney test). (**C**) ChIP-qPCR of RNAPII (8WG16 antibody) before and after E2 stimulation (30 min) and with or without H2A.Z depletion by shRNA. Five loci of both ERα w/ H2A.Z (loci (A) to (E)) and ERα w/o H2A.Z (loci (F) to (J)) were studied. The results are normalized to shCTL +E2 and represent the mean ± SEM of two independent biological replicates. The coordinates of each locus as well as the sequences of the primers are available in the Supplementary Table 3. (**D**) RT-qPCR analyses using the same conditions and primers than panel (C). The expression levels are relative to the expression of *RPLP0* and are normalized to shCTL -E2. The results represent the mean ± SEM of four independent biological replicates. (**E**) Normalized RAD21 signal distribution at intergenic ERα-active enhancers in the presence of E2. The signal of RAD21 is significantly higher in ERα w/ H2A.Z (*P*-value < 0.0001, Mann-Whitney test). (**F**) Same analysis as (C), but using RAD21 antibody.

Recently, RNA polymerase II (RNAPII) binding has been found at a subset of enhancers and shown to drive the transcription of a new class of noncoding RNA, the enhancer-associated RNAs (eRNAs) (65–67). Although the functions of eRNAs remain poorly understood, it is generally thought that their expression correlates with enhancer activity. Indeed, recent studies support a role of eRNAs in the regulation of expression of adjacent protein-coding genes by establishing chromatin accessibility and influencing RNAPII occupancy at promoters (68), and by promoting specific enhancer-promoter interactions via chromatin loops (69,70). At ERα enhancers, transcription of eRNAs has been shown to be mainly upregulated following E2 stimulation (67,70–71) and to be associated with H3K4me1 and accessible chromatin state (71), as well as with H3K27ac (70). Since H2A.Z has been shown to help the recruitment of RNAPII at promoters (34,51), we hypothesized that H2A.Z could be important for enhancer functions by recruiting RNAPII also responsible of eRNAs transcription at enhancers. We first sought to address whether ERα w/ H2A.Z exhibits eRNAs production using global run-on-sequencing (GRO-seq) datasets (71), which report and quantify the genome-wide localization of nascent RNAs associated with transcriptionally engaged RNA polymerases (72). This analysis was limited to enhancers located in intergenic regions to avoid contamination by mRNA transcripts. Interestingly, transcription at intergenic ERα w/ H2A.Z enhancers increases after 40 min of E2 stimulation (Figure 6A and Supplementary Figure S11A and B). In contrast, a basal level of eRNAs is observed at ERα w/o H2A.Z, seemingly independent of E2 treatment. We thus isolated E2-regulated genes specifically associated with ERα w/ or w/o H2A.Z to explore whether the increase of E2-inducible eRNA levels at enhancers enriched for H2A.Z are linked to a similar modulation of gene expression. Indeed, the induction of the predicted E2-target genes after 40 min of E2 stimulation is significantly higher for the group of genes associated with ERα w/ H2A.Z than those associated with ERα w/o H2A.Z (Figure 6B and Supplementary Figure S11C and D).

We then asked whether H2A.Z is directly involved in RNAPII recruitment and eRNAs transcription at these enhancers. To address this issue, we first investigated by ChIP-qPCR the binding of RNAPII at 10 ERα w/ or w/o H2A.Z loci using two different RNAPII antibodies. RNAPII is recruited at both groups and as expected its level increases upon E2 stimulation (Figure 6C and Supplementary Figure S12A and B). A small hairpin RNA (shRNA)-mediated depletion of H2A.Z (Supplementary Figure S12C and D) results in a decrease of RNAPII recruitment to ERα w/ H2A.Z in response to E2 (Figure 6C and Supplementary Figure S12A and B), with no significant alteration of ERα binding (Supplementary Figure S12E). The loss of H2A.Z at chromatin following its depletion is mostly compensated by a gain of canonical H2A (Supplementary Figure S12F) and thus does not seem to induce nucleosome depletion. Rather, this result could indicate an increase in nucleosome stabilization or deposition or a decrease in nucleosome removal, and thus a loss of chromatin accessibility. Using RT-qPCR assays, we next addressed whether eRNAs production is affected by the loss of RNAPII at ERα w/ H2A.Z enhancers following H2A.Z depletion. As shown in Figure 6D, E2-regulated transcription at ERα w/ H2A.Z is abolished in absence of H2A.Z. Importantly, RNAPII recruitment as well as eRNAs transcription are not affected by H2A.Z depletion at ERα w/o H2A.Z (Figure 6C and D and Supplementary Figure S12A and B). Furthermore, we observed that the transcription at ERα w/ H2A.Z seems more E2 inducible than at ERα w/o H2A.Z, confirming the results reported in Figure 6A. Unfortunately, the presence of H2A.Z at the TSS of genes associated with ERα w/ H2A.Z and w/o H2A.Z (Figure 2D and Supplementary Figures S11D, S13 and S14) prevents to investigate the effect of its depletion on mRNA transcription. Indeed, such an experiment would not allow to be able to conclude whether the effect is related to a lack of H2A.Z at enhancer or at TSS.

Li *et al*. (70) reported that eRNA transcripts and the cohesin complex are implicated in enhancer-promoter chromatin interactions at two loci. Indeed, eRNAs are required for RAD21 recruitment (70), a component of the cohesin complex involved in chromosomal interactions (45,73), and the formation and/or maintenance of the chromatin loops (69,70). The finding that H2A.Z is necessary for RNAPII recruitment and transcription at enhancers raises the possibility that H2A.Z depletion may also affect the effectors of chromatin loops. We observed that the RAD21 signal (45) is significantly higher at intergenic ERα w/ H2A.Z (Figure 6E and Supplementary Figure S15A–C), suggesting more chromatin interactions. Moreover, intergenic ERα w/ H2A.Z are more involved in chromatin looping than ERα w/o H2A.Z, as defined by the significantly higher signal of RNAPII-dependent chromatin interactions analysis by paired-end tag sequencing (ChIA-PET (74)) generated on unsynchronized cells exposed to E2 naturally present in the culture media (Supplementary Figure S15D and E). Importantly, as for the RNAPII, the H2A.Z depletion causes a decrease of E2-dependent recruitment of RAD21 specifically at H2A.Z-enriched enhancers (Figure 6F and Supplementary Figure S12G). Taken together, these data suggest that H2A.Z is an important regulator of enhancer functions at a subgroup of active enhancers by establishing and maintaining a chromatin environment required for RNAPII recruitment, eRNAs transcription and enhancer–promoters interactions, all essential attributes of enhancer activity.

## DISCUSSION

**Figure 7.**
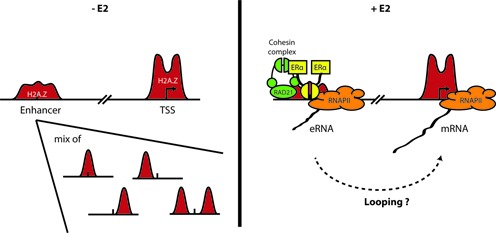
A schematic summary of the H2A.Z-dependent regulation of enhancer activity.

In this study, we provide evidence for H2A.Z influence at inducible enhancers by exploring its relationship to the local chromatin environment and structure. A schematic summary of our principal mechanistic conclusions at ERα w/ H2A.Z enhancers is presented in Figure 7. We uncovered that H2A.Z is not present at all enhancers but rather marks the most active and regulated ones, defined as having a significantly higher level of ERα (Figure 1B) and DNase I hypersensitivity (Figure 4A), higher inducible expression of eRNAs and their putative target genes (Figures 6A and B), as well as higher presence of chromatin loops, inferred by RAD21 and ChIA-PET of RNAPII signals (Figures 6E and Supplementary Figure S15). These conclusions are derived from the direct comparison between H2A.Z-occupied and -unoccupied enhancers, both harboring the H3K27ac mark, and thus are unbiased in regards to their activity. Moreover and importantly, our analyses emphasize the importance of considering enhancers as a heterogeneous population. Our results also suggest that H2A.Z could help the detection of the most relevant active enhancers.

An important finding of the present study is that our analyses support a role for H2A.Z in promoting transcription through the recruitment of RNAPII at enhancers, the transcription of eRNAs and the possible formation and/or stabilization of the enhancer–promoter interactions (Figure 7). Indeed, we demonstrated here for the first time that H2A.Z is necessary to recruit RNAPII and RAD21 and to express eRNAs (Figure 6C,D,F) for a group of active enhancers where H2A.Z presence is cell-type specific (Figure 5E). Importantly, these recruitments and their concomitant production of eRNAs are E2 inducible. Our results highlight the role of H2A.Z as a master regulator of enhancer functions, but do not exclude the implication of other effectors such as the Mediator complex (69,73). Also, the sequence of events cannot be clearly established from our results and will require further investigations. Nonetheless, in line with the results obtained by Li *et al*. (70), the most likely mechanism is that the decreased recruitment of RNAPII caused by H2A.Z depletion prevents the inducible transcription of eRNAs and thus the association of the cohesin complex and the formation and/or the stabilization of chromatin loops. Interestingly, since RNAPII and active transcription was detected at enhancers and associated with the transcription level of the surrounding protein-coding genes (65,66), an emerging hypothesis for enhancers function is their role in RNAPII delivery via chromatin loops. The fact that H2A.Z is essential to the inducible recruitment of RNAPII at enhancers (Figure 6C) and promoters (34,51) provides support for this model, but further experiments will be required for its confirmation. Finally, this proposed mechanism might be specific to H2A.Z-enriched enhancers, since the inhibition of chromatin looping between enhancers and their target promoters was not generalized to all tested loci following inhibition of eRNA production (71), and neither the inhibition of RAD21 recruitment following eRNA depletion (68). Regarding the ERα w/o H2A.Z enhancers, another mechanism could be implicated since eRNA production seems rather constitutive (Figure 6A), and RNAPII and RAD21 recruitments are independent of H2A.Z (Figure 6C,F). Finally, how H2A.Z helps the recruitment of RNAPII is yet unknown, but it could be related to its effect on the chromatin environment.

A substantial proportion of distal ERα-BS or distal H2A.Z-enriched regions contain potential unannotated promoters (Supplementary Figure S2). Interestingly, a recent ENCODE study identified an extensive number of unannotated TSS by studying the relationship between DHS and H3K4me3 patterns at well-annotated TSS (12). Since these distal-contaminated regions may be enriched by both H3K4me3 and H2A.Z, particular attention should therefore be taken in the interpretation of the role of H2A.Z at enhancers in absence of other measure than known gene annotations used to discern enhancers from promoters. This prediction is supported by a recent study, where ∼60% of the H2A.Z-enriched p300-intergenic sites reported as enhancers are associated with H3K4me3 and have been found to be associated with housekeeping functions, while those not enriched for H3K4me3 are associated with system development and differentiation, more related to cell-type-specific gene expression, and thus to enhancer functions (29).

Unexpectedly, we found that H2A.Z does not influence the local nucleosome positioning at ERα-active enhancers. Indeed, as shown in Figure 3, none of the nucleosome patterns obtained by mixing ERα w/ and w/o H2A.Z enhancers are enriched for regions containing H2A.Z. Hence, when clustered separately, both ERα w/ and w/o H2A.Z contain a similar mix of nucleosome organization classified as centered or side. The observed asymmetry in the nucleosome positioning, histone modification deposition and H2A.Z occupancy around ERα-active enhancers, as well as the shapes of the predominant clusters are in agreement with results obtained by others (19). However, our study includes new observations that provide a more complete understanding of the relationship between H2A.Z or histone marks and nucleosome positioning around TF-BS in an inducible model. Using E2-synchronized cells and ChIP at nucleosome resolution, rather than a population of unsynchronized cells and random shearing by sonication, we clarified that nucleosome occupancy profiles colocalize with H2A.Z or histone marks profiles, and that H2A.Z colocalize with H3K4me1 and H3K27ac at ERα enhancers (Figure 3D and Supplementary Figures S6B, S7B and S8). Other investigators have observed a broad histone peak centered over the ERα-BS and have suggested that ERα binding is not associated with change in nucleosome occupancy directly over the binding site itself (24). This conclusion has been essentially inferred from average signal profiles of H3K4me2 over all distal ERα-BS or by the specific search of a symmetric pattern, which ignores the diversity in nucleosome organization as well as the individual signal level. Indeed, the average signal profile of H3K4me1 around distal ERα summits gives the centered profile previously described for H3K4me2 (Supplementary Figure S16A) which obscures the diverse observed scenarios in nucleosome positioning patterns (Figure 3D, also obtained using H3K4me2 from (24), Supplementary Figure S7A) and masks the combination between histone marks (Supplementary Figure S3A). Alternatively, some studies have reported dynamism in nucleosome positioning or occupancy (20–22), including nucleosome-containing H2A.Z (59,75). These reported discrepancies, in addition to be frequently caused by methodology issues as explained previously, could be a consequence of differences between differentiation and inducible models, or TSS and enhancer localization of H2A.Z, or could also arise by the contamination of potential H2A.Z-enriched enhancers by unannotated TSS. Using a pattern discovery method (19), we have clarified that ERα binding to active enhancers is not associated with changes in nucleosome positioning, regardless of the presence of H2A.Z, and occurs in a mixed scenario of local nucleosome positioning.

We observed a persistence of H2A.Z and DNase I signal levels at ERα w/ H2A.Z from basal to E2-stimulated states (Figures 1A and 3D, and Supplementary Figures S3C, S7B and S9A) indicating that ERα is mainly targeted at pre-existing accessible loci. However, the absence of an apparent drastic dynamism in these levels and in nucleosome positioning does not necessarily mean that chromatin structure is static. Indeed, the enrichment of H2A.Z, the higher DNase I signal and the enrichment of H3K122ac (Figure 4), which disturbs histone-DNA contact and thus the nucleosome dynamics (53,76), suggest that the chromatin environment is highly dynamic at ERα w/ H2A.Z. Consequently, the higher signal of ERα in the centered category of ERα w/ H2A.Z (Figure 3C, representing the predominant configuration (Figure 3B)) could imply that ERα competes with dynamic nucleosomes for binding. Hence, a given region can either be occupied by H2A.Z-containing nucleosome or by ERα. However, ChIP experiments provide a static snapshot and thus limit the conclusions we can reach with our study. Nonetheless, some evidence in the literature support the hypothesis of a rapid and dynamic transition between ERα and H2A.Z-containing nucleosomes (16–17,53,77–78). For instance, a higher turnover rate has been observed when H3.3 is associated with H3K4me1, H3K27ac and H2A.Z (78). Since H2A.Z deposition at distal DHS occurs almost exclusively with H3.3 in HeLa cells (17), it is fair to assume that H3.3 should be enriched at the majority of ERα w/ H2A.Z and that they could work together to destabilize nucleosomes. In addition, H3K122ac is already present at ERα w/ H2A.Z in absence of E2 (Figure 4B) and its level has been shown to increase following E2 stimulation at the enhancer of *TFF1* (53). Thus, the dynamic and highly accessible chromatin environment at H2A.Z-enriched enhancers could provide the permissive landscape to keep those regions ready in absence of E2, and prime a rapid and dynamic transition between nucleosomes and ERα local occupancies in presence of E2.

Another hypothesis that is not mutually exclusive, is that ERα binds nucleosomal DNA. One way for TFs to transiently gain access to nucleosomal DNA is via nucleosome breathing, the partial DNA unwrapping and rewrapping, that can be rapid and spontaneous (79–81) or mediated by DNA remodeling proteins. Histone modifications within the globular domain of histones affect this dynamism and can shift the equilibrium toward the partially unwrapped state. Indeed, H3K122ac together with H3K115ac has been suggested to be implicated in the maintenance of a partially unwrapped nucleosome (76), supporting the unwrapping hypothesis for ERα w/ H2A.Z. Moreover, it has been proposed that acetylation of the N-terminal tail of H2A.Z could weaken the contacts between the tail and DNA and thus increase the accessibility of DNA (28). Interestingly, the acetylated form of H2A.Z is already enriched in ERα w/ H2A.Z in unstimulated state (Supplementary Figure S5D), providing an additional argument to the unwrapping hypothesis.

Regarding the active ERα w/o H2A.Z enhancers, the accessibility, even if lower than w/ H2A.Z (Figure 4A), could be a consequence of constitutive eRNAs transcription (Figure 6A), or the presence of other TF-binding events, other histones variants or other histone marks not tested in this study. For instance, H3K56ac has been shown to facilitate DNA unwrapping and TF-binding within nucleosomes (76,82–84). Since H3K56ac antagonizes H2A.Z deposition (85), it could be an interesting endeavor to explain the centered category observed in ERα w/o H2A.Z enhancers. Finally, the method used in this study, i.e., the single-end sequencing of mononucleosomal fragments from a population of cells and the clustering of signal shapes, can not capture the dynamical binary state of the exchange between ERα and nucleosomes, neither the hardly detectable subtle changes induced by partial DNA unwrapping. Future studies would benefit from the paired-end sequencing of subnucleosomal particules (86) to explore the potential local conformational variations or transitions of the nucleosome at enhancers, containing or not H2A.Z, through a cell population or from a genomic approach at single-cell level.

The most intriguing remaining question is why only a subset of ERα-active enhancers is occupied by H2A.Z. The close relationship between H2A.Z deposition and DNA methylation level that we identified at ERα-active enhancers (Figure 5), which is in-line with previous findings at TSS and in gene bodies (58,59), suggests that the methylation status of the underlying DNA could be a part of the answer. In fact the conservation of the un/low-methylated status of enhancers seems to be essential since it is maintained even in hypermethylated gene bodies of expressed genes (62). Moreover, considering that the level of H2A.Z and the hypomethylated state are not conserved in normal HMEC (Figure 5E), and that the ‘high-confidence’ hypomethylated ERα w/ H2A.Z enhancers not prebound by ERα in absence of E2 is associated with genes with functional annotations related to cell growth, invasion and migration (Supplementary Table 2), it is tempting to speculate that this group of enhancers results from an accumulation of epigenetic deregulations during breast carcinogenesis, such that a deregulation in the deposition of H2A.Z at enhancers could affect their DNA methylation patterns and vice versa. Indeed, H2A.Z is overexpressed in breast cancer and is associated with high-grade/metastasis tumors (87,88) and decreased patient survival (87), and a massive loss of DNA methylation has been observed in breast cancer cell lines compared with HMEC (89,90), especially in CpG poor regions (89) or intergenic regions (90), both being a characteristic of enhancers. Moreover, epigenetic alteration by the reshuffle deposition of H3K4me1 has been recently demonstrated as a thread of colorectal carcinogenesis (4), and H2A.Z redistribution from TSS to gene bodies and opposite changes in DNA methylation status were observed during tumorigenesis (59). Importantly, a recent study supports the alteration of methylation at enhancers as potential perturbator of transcriptional programs (91). For instance, the altered transcriptional program observed in MCF-7 cells compared with HMEC correlates with an altered methylation state at enhancers. Thus many epigenetic alterations in the course of carcinogenesis could lead to a redistribution of H2A.Z. Our results of opposite patterns for H2A.Z and DNA methylation, and for DNA methylation and chromatin accessibility, as well as the close relationship between H2A.Z and chromatin accessibility were somewhat expected based on the known distribution of these epigenetic features. However, our results reveal a complex and new interlink between those three players and TF occupancy at active enhancers and point toward H2A.Z as the missing epigenetic link. As a consequence, and in-line with the striking functional link between the presence of H2A.Z at enhancers, RNAPII recruitment, eRNAs transcription and its potential involvement in chromatin loops with promoters (Figure 6), a deregulation in H2A.Z deposition might lead to the spurious recruitment of RNAPII at inappropriate regions in the genome, and thus, to transcriptional disturbances. Future studies aimed at characterizing epigenetic changes in cancer, for instance whether environmental carcinogens, cancer treatments and drug resistance affect H2A.Z distribution, will be highly informative.

## ACCESSION NUMBER

Sequencing data have been deposited in the NCBI's Gene Expression Omnibus (GEO) (92) under GEO Series accession number GSE57436 (http://www.ncbi.nlm.nih.gov/geo/query/acc.cgi?acc=GSE57436).

## SUPPLEMENTARY DATA

Supplementary Data are available at NAR Online.

SUPPLEMENTARY DATA
