# Supplementary material for: The histone variant H2A.Z is an important regulator of enhancer activity
Source: Nucleic Acids Res. 2015 Aug 28;43(20):9742–56. doi: 10.1093/nar/gkv825 (PMC4787790; doi:10.1093/nar/gkv825)
Supplement: SUPPLEMENTARY DATA [file supp_43_20_9742__index.html]

The histone variant H2A.Z is an important regulator of enhancer activity — SUPPLEMENTARY DATA 

# The histone variant H2A.Z is an important regulator of enhancer activity

## SUPPLEMENTARY DATA

- SUPPLEMENTARY DATA
- SUPPLEMENTARY DATA
- SUPPLEMENTARY DATA
- SUPPLEMENTARY DATA
- SUPPLEMENTARY DATA
- SUPPLEMENTARY DATA
