# Supplementary material for: The histone variant H2A.Z is an important regulator of enhancer activity
Source: Nucleic Acids Res. 2015 Aug 28;43(20):9742–56. doi: 10.1093/nar/gkv825 (PMC4787790; doi:10.1093/nar/gkv825)
Supplement: SUPPLEMENTARY DATA [file supp_gkv825_nar-00353-x-2015-File011.pdf]

Supplementary Material

Methods

Cell culture and treatments

The MCF-7 cells were grown in DMEM (Wisent) supplemented with 10% fetal bovine serum (FBS) and antibiotics. Before experiments, the MCF-7 cells were hormone-deprived for three days in DMEM without phenol red (Wisent) supplemented with 5% charcoal-stripped FBS. The cells were treated with 100 nM of 17β-estradiol (E2, Sigma) or with the vehicle (EtOH) for 30 minutes. The cells were mycoplasma free (routinely tested). The shRNA directed against H2A.Z was transfected into MCF-7 as described previously (1). Western blotting were done to confirm effective shRNA-mediated depletion of H2A.Z (a typical result is shown in Supplementary Fig. S12C).

Antibodies

The following antibodies were used: ERα (Santa Cruz Biotechnologies HC-20 sc-543, lot #B1413); H2A.Z (Abcam ab4174, lots #808425, #GR15952-1 and #GR15952-3); H3K27ac (Abcam ab4729, lot #961080); H3K4me1 (Abcam ab8895, lot #899421); H3K4me3 (Active Motif 39159, lot #01609004); H2A (Abcam ab18255, lot #GR145536-1); RPB2 (Abcam ab10338, lot #GR19662-14); RNA polymerase II CTD repeat YSPTSPS (8WG16, Abcam ab817, lot #GR153063-402); and Rad21 (Abcam ab992, lot #GR12688-10).

Isolation and immunoprecipitation of cross-linked mononucleosomes (ChIP-MNase)

Since the nucleosomes containing both H2A.Z and H3.3, which are mainly present at enhancers, are subjected to disruption and are lost during cell preparation if they are not cross-linked (2–4), MCF-7 cells were cross-linked 10 min at room temperature (1% formaldehyde in 1X PBS pH 7.4). ChIP assays were performed as described previously (5), except for the chromatin preparation. Briefly, permeabilized cells were incubated 2 min at 37°C in digestion buffer (50 mM Tris-HCl pH 8.0, 1 mM $CaCl_2$, 0.2% Triton and protease inhibitors), before the addition of Micrococcal Nuclease (MNase, USB Corporation) 5 min at 37°C. Then the digestion reactions were stopped (5 mM EDTA, 10 mM Tris-HCl pH 8 and 0.5% SDS and protease inhibitors) and chromatin sample were briefly sonicated to fragment insoluble chromatin from the pellet without affecting the fragmentation of mononucleosomal fragments (6). No visible pellet remained after a centrifugation at 15,000 g at 4 °C for 10 min. The digestion and the full extraction of chromatin were verified on gel electrophoresis prior to the immunoprecipitation of the chromatin. Two biological replicates were done for each ChIP-sequencing experiment and for each condition. In addition, the proper recruitment of ERα at some known enhancers following E2-stimulation was verified for each ChIP-sequencing experiment.

The MNase digestion is a critical parameter, particularly to compare two conditions, i.e., with and without E2. Thus, titrations with increasing amount of MNase were done in order to insure uniformity and repeatability of the digestion and to minimize potential artifacts caused by overtrimming or undertrimming DNA. The 2100 Bioanalyzer instrument (Agilent Technologies) was used to resolve DNA fragments following MNase digestion, reaching a precision that conventional gel electrophoresis cannot. It separates DNA fragments with a resolution as good as 5 bp and allows the direct comparison of samples (see Supplementary Fig. S17A for a typical MNase titration experiment). The proportion of mononucleosome as well as the length of mononucleosome DNA fragments were computed for three replicates (Supplementary Fig. S17B). The amount of MNase that gives 80% of mononucleosomes and an expected mononucleosome length between 145-150 bp (150 U) was selected for subsequent MNase-ChIP-seq experiments.

Preparation of the sequencing libraries

The sequencing libraries were performed according to the Illumina library preparation protocol except for the size-selection of DNA fragments and for DNA recovery that were executed as described previously (7). Quant-iT PicroGreen dsDNA Assay Kit (Invitrogen) was used to quantify ChIP-DNA and 10 ng was used for the preparation of the sequencing libraries. The size-selection of nucleosome fragments, to exclude subnucleosomal and polynucleosomal particules, was executed in parallel with the preparation of libraries and was performed using solid-phase paramagnetic beads technology (AMPure XP PCR Purification systems, Beckman Coulter), since it allows a more precise size-selection of the DNA fragments than conventional gel electrophoresis and minimizes loss of sample, which is particularly relevant for the dilute samples frequently obtained after ChIP experiments. It was thus used for all the DNA-recovery steps of the libraries preparation as well as to eliminate potential primer dimers or primers and linkers in excess (shorter than the libraries of nucleosome fragments) after the final amplification of the libraries. Importantly, this final step was monitored by qPCR and stopped in the exponential-phase to avoid PCR-duplicates. The quantification of the final libraries, the absence of primer-dimers as well as the validity of the size-selection were determined using the 2100 Bioanalyzer instrument (Agilent Technologies). To ensure reproducibility, two biological replicates of every mark or H2A.Z or control input were single-end sequenced with 40 nucleotides reads using an Illumina HiSeq 2000 platform by the MIT MicroBioCenter.

RNA preparation, RT-qPCR and microarray

For microarray analysis, total RNA was extracted from MCF-7 cells (-/+ 100 nM E2, 4h) using the GenElute Mammalian Total RNA Miniprep kit (Sigma), then RNA was reverse transcribed using M-MLV reverse transcriptase (Promega) according to manufacturer's instructions. Before microarray analysis, samples were subjected to qPCR to test the appropriate induction of known E2-target genes and the quality and integrity of RNA samples were verified using the 2100 Bioanalyzer instrument (Agilent Technologies). Two biological replicates of microarray analysis were performed using the Human HT-12 Expression BeadChips (Illumina) at the McGill University and *Génome Québec Innovation Centre* (Montréal, QC, Canada). For the analysis of transcription at enhancers, total RNA, including small RNAs, was isolated using the Direct-zol RNA MiniPrep (Zymo Research) with an additional step of DNase I digestion.

Data analysis

Processing of ChIP-Seq libraries

The alignment of sequencing reads onto the human genome (Build 36.1, hg18, Mar. 2006) was done using the software Burrows-Wheeler Aligner (BWA, version 0.6.1, (8)) with default parameters. Above 96% of the reads per sample were mappable and ∼80% had a Phred Mapping Quality Score above 10 (filtered using Samtools, version 0.1.18 (9)). The Picard command-line tools (version 1.64, http://picard.sourceforge.net) was then used to remove duplicates (above ∼80% of the filtered data were kept). Since the Pearson correlation coefficient, calculated in 2 kb bins containing the number of reads across the whole genome, was above 0.9 between each biological replicated (except inputs) (Supplementary Fig. S1), both replicates were combined for subsequent analyses. Significantly enriched regions were detected using MACS (version 1.3.7, (10)) taking control input into account and using the default parameters except for the "band width" that was set to 150 bp (–bw=150) according to our experimental data and "keep duplicates" that was set to "all" (–keep-dup=all). The signal files were then normalized (see section "Nucleosome organization analysis").

Public data set used in this study and processing

The following data sets of processed enriched regions were directly used as is: FOXA1 and AP-2γ (GSE23852 (11)), PBX1 (GSE28007 (12)), ChIA-PET of ERα (GSE18046 (13)), ERα-consensus binding sites (11), p300 or CBP (E-MTAB-785 (14)), ERα-EGF cistrome (GSE26081 (15)).

The Sequence Read Archive (SRA) of ERα and respective control input (ERR011973, ERR011978, ERR011971, ERR011972 of the series GSE25021 (16)) were processed the same way than our ChIP-seq samples, except that after visual inspection and exploration of the p-value, the false discovery rate and the fold enrichment a cut-off was applied on the first quartile of the fold enrichment since it appears as the most effective way to eliminate the poor quality peaks.

The DNA methylation data sets used in this study were generated by Reduced Representation Bisulfite Sequencing (RRBS) from ENCODE by the Myers's lab at the Hudson Alpha Institute for Biotechnology (http://genome.ucsc.edu/cgi-bin/hgFileUi?db=hg19&g=wgEncodeHaibMethylRrbs, (17)). Because the results obtained were found consistent through the six replicates available, we limited the representation of the results to only the Stanford's replicates. When less than 25% of a particular CpG was found methylated, the CpG was classified as unmethylated, from 25% to 75% as partially methylated, and when greater than 75% as fully methylated. The partially and fully methylated CpG were considered methylated for the Figures 5B-C, however, the full results can be found in the Supplementary Figures S10C,I.

The DNase I sensitivity were generated by the University of Washington group (http://genome.ucsc.edu /cgi-bin/hgFileUi?db=hg19&g=wgEncodeUwDnase). The H2A.Z signals from other cell lines tested were generated by the Broad Institute group and the Bernstein lab at the Massachusetts General Hospital/Harvard Medical School (http://genome.ucsc.edu/cgi-bin/hgFileUi?db=hg19&g=wgEncodeBroadHistone). These files were downloaded from the ENCODE portal in the BAM format (Binary Alignment/Map) and processed the same way than our ChIP-seq samples. The signals files were then uniformly processed and normalized using Wiggler tool (18) (see section "Nucleosome organization analysis").

The ChIP-seq of H3K122ac and control from the series GSE35954 (SRR863216, SRR863218, SRR863 219, SRR863220, (19)), the ChIP-seq of RAD21 from the series GSE25021 (ERR011976, ERR011977, (16)) and the ChIP-seq of H3K4me2 from the series GSE33216 (SRR358668, SRR358669 (20)) were downloaded in the SRA format, then treated as our ChIP-seq experiments and normalized using Wiggler tool (18) (see section "Nucleosome organization analysis").

The signals of Chromatin Interaction Analysis Paired-End Tags (ChIA-PET) of RNA polymerase II generated by Genome Institute of Singapore (http://genome.ucsc.edu/cgi-bin/hgFileUi?db=hg19&g=wgEncode GisChiaPet, (21)) were downloaded in WIG format from the ENCODE portal. The BEDTools subcommand "complement" (version 2.16.2, (22)) was used to set the absence of signal to zero.

The processed signals of GRO-seq data sets (series GSE43836, (23)) were downloaded in BED format, then extended to the size of the sequencing libraries (100 bp) and normalized to obtain strand specific bedgraph files using, the subcommands "slop" and "genomecov" of BEDTools (22). The analyses were done using the two biological replicates, but given the results were highly similar, only replicate 1 was kept for final representation. The processed file from the series GSE27463, representing the change in the expression of annotated transcripts (at 0, 10, 40 and 160 min), was also downloaded and used in the Figure 6B.

For all the analyses involving processed files originally generated using the hg19 assembly, the ERα coordinates or the processed files coordinates were converted, either to hg19 or hg18 using the liftOver tool from UCSC (http://genome.ucsc.edu/cgi-bin/hgLiftOver).

Genomic regions assignment

Distal regions were defined as being at more than 3 kb of known TSS (from a combined list of the hg18 RefSeq genes and UCSC genes, downloaded from http://genome.ucsc.edu/cgi-bin/hgTables), proximal regions being therefore within 3 kb of TSS. Similarly, genic and intergenic regions were defined as being within or outside the hg18 combined genes list from RefSeq and UCSC genes.

To associate the ERα-summits to potential gene targets, a regulatory domain for each gene was defined by a basal domain (-5 kb/+1 kb from TSS) and an extension domain up to the basal domain of the nearest genes within 1 Mb using the "createRegulatoryDomains tool" of the GREAT source code (24), then the overlap between those domains and the ERα-summits was computed. Depending on the analyses, either the gene names or the unique TSS coordinates were used. In this later case, redundant TSS, having the same coordinates but different names, were removed.

The "high-confidence" ERα un/low-methylated enhancer network showing no binding of ERα in the absence of E2 (w/ and w/o H2A.Z) used in the GO analysis (Supplementary Table 2) were defined as ERα for which all overlapping CpG were in the same bracket on DNA methylation level (unmethylated, partially methylated or fully methylated) through both replicates of Stanford's data sets. The proportions of ERα-BS in each category of the list of "high-confidence" are similar to those observed using the complete lists of ERα w/ or w/o H2A.Z for both replicates.

The subcommand "intersect" of BEDTools (22) was used for all the analyses requiring the overlap or the substract of two files of genomic coordinates.

K-means Clustering

The K-means clustering of the read density of the chromatin mark signals centered on TSS-distal or TSS-distal non-promoter ERα-summits or TSS-proximal/distal H2A.Z regions were performed using seqMINER tool (version 1.2, (25)). A window of 2 kb subdivided in 50 bp bins was used. All reads were extended at 150 bp according to the strand information and the ranked-based normalization was used. In each case, we varied the number of clusters from 2 to 10 at least 10 times, and choose the minimal number of stable clusters by which new clusters represent subgroup of existing clusters.

Rationale for the exclusion of H3K4me3-enriched regions

There are discrepancies in the literature regarding whether or not H3K4me3 could be found at enhancers (26–31). Since H2A.Z correlates with H3K4me3 at promoters (26, 28, 30, 32), it is thus crucial to first clarify this in our data in order to remove potential unannotated promoters and delineate the specific role of H2A.Z at enhancers. When H3K4me3 datasets were included in the K-means clustering on distal ERα-BS, we observed that potential ERα-active enhancers w/ H2A.Z were subdivided for the presence and absence of this histone modification (cluster 1 and 2, Supplementary Fig. S2C). To evaluate the possibility that these H3K4me3-enriched regions could be potential unannotated promoters, we calculated the proportion of each cluster overlapping CpG islands, as well as analyzed the probability for these sequences to be a TSS using Profisi (33). As shown in Supplementary Figure S2C, the cluster 1 (C1) containing ERα/H2A.Z/H3K4me3 co-enriched regions are likely to be unannotated promoters, whereas the cluster 2 (C2) containing ERα/H2A.Z without H3K4me3 are not. We also used K-means clustering centered on H2A.Z enriched regions distal or proximal to TSS in order to evaluate the proportion of those regions enriched for H3K4me3 (Supplementary Figs. S2A,B). As expected, we observed that proximal H2A.Z/ERα co-occuring regions have a chromatin state distinct from distal regions. At proximal regions,

H2A.Z/ERα are associated with high level of H3K4me3 and low level of H3K4me1, whereas at distal regions, H2A.Z/ERα are mostly associated with a strong level of H3K4me1 and a low level of H3K4me3. Moreover, the three proximal clusters (P1 to P3) have promoter properties, while only the distal cluster 1 (D1) containing a high level of H3K4me3 could represent uncharacterized TSS, in line with a recent ENCODE study reporting an extensive number of unannotated TSS (34). As a consequence, we removed from the ERα-BS the 2905 regions overlapping H3K4me3-enriched regions identified by MACS (see above) for all our analyses.

Gene expression analysis

Gene were split in four categories based on our expression results following E2-stimulation: up-regulated, down-regulated, expressed but not affected by E2-treatment and not expressed. A regulatory domain for each gene was defined as described above and a relative enrichment, against a random distribution (100 iterations of 60,000 distal genomic regions), was calculated for each ERα-group to generate the results presented in Figure 1C. Complete results of our gene expression analyses are available in Supplementary Tables 4 and 5.

Average signal profiles

The average signal profiles were generated using the Versatile Aggregate Profiler tool (VAP, version 1.0.0) (35). The following parameters were used: the Annotation (Fig. 2D and Supplementary Fig. S11D) or Coordinate (Fig. 6 and Supplementary Figs. S11A-C) modes, the absolute representation, the txStart (Fig. 2D and Supplementary Fig. S11D) or ERα summits (Fig. 6 and Supplementary Figs. S11A-C) as reference point, the 5' coordinates of reference feature as boundary, 1 reference point, 100 windows of 50 bp (Fig. 2D and Supplementary Fig. S11D) or 10 bp (Fig. 6 and Supplementary Figs. S11A-C) in the upstream and downstream blocks, the mean aggregate value, and no smoothing.

Nucleosome organization analysis

The Wiggler tool (version 1.0, https://code.google.com/p/align2rawsignal/, (18)) was used to uniformly process and normalize the MNase ChIP-seq data using the default parameters except for the fragment length that was set to 147 bp (reads are shifted by 147/2 from 5' to 3'), the smoothing window that was set to 120 and the smoothing kernel that was set to triweight. This tool normalized the genome-wide signal at each position as a fold-change (the observed fragment count over the expected fragment count per position, i.e, if all the uniquely mappable reads are uniformly distributed over all uniquely mappable locations in the genome). In the resulting normalized signal, the value is "0" at mappable locations with zero read, while the value is "N/A" at not uniquely mappable locations. The extractSignal tool (version 1.0, https://code.google.com/p/extractsignal/) was used to extract the signal 150 bp on each side of ERα-summits (bins of 10 bp). Then, CAGT (Clustered AGgregation Tool, version 127, https://code.google.com/p/cagt/, (36)) was used to performed clustering of ERα-active enhancers (mix of ERα w/ and w/o H2A.Z) or individual group, according to the shape of associated H3K4me1, H2A.Z or MNase-sequencing signals. The following parameters were used: "lowSignalCut", 0.05, "lowSignalPrc", 99, "lowVarCut", 0.0001, "distance", sqeuclidean, "avgFun", mean, "k", 40, "start", plus, "replicates", 1000, "maxiter", 500, "merge", true, "mergeK", 1, "mergeDist", 8, "flip", true.

Classification of the nucleosome shapes

For the Figures 3B-C and S6A, the nucleosome shapes of ERα w/ and w/o H2A.Z were independently classified into 3 categories, based on the H3K4me1 signal at ERα-summit as well as visual inspections. For

each shape, the mean signal of H3K4me1 at ERα-summit was normalized on the mean signal of input over the 300 bp. The side category was defined as below or equal to 5, the centered category as greater than 7 and the ambiguous category as the boundaries of the thresholds. Individual snapshots of each category were used to validate the thresholds. DANPOS (Dynamic Analysis of Nucleosome position and Occupancy by Sequencing, version 2.1.0, http://code.google.com/p/danpos/, (37)) was used to define the preferential position of nucleosome containing H3K4me1 using default parameters except for the following: the smooth width to 0, a step size of 1 bp in the final wig files, the quantile normalization method, an intensity cut-off for nucleosome calling of 6 and a cut-off of 1 for adjusting the clonal signal. Then for each category, the distance between ERα and the nearest nuclesome summit was computed using the subcommand "closest" of BEDTools (22).

References

[1] Gévry, N., Hardy, S., Jacques, P.-E., Laflamme, L., Svotelis, A., Robert, F., and Gaudreau, L. (2009) Histone H2A.Z is essential for estrogen receptor signaling. *Genes Dev,* **23**(13), 1522–1533.

[2] Jin, C. and Felsenfeld, G. (2007) Nucleosome stability mediated by histone variants H3.3 and H2A.Z. *Genes Dev,* **21**(12), 1519–1529.

[3] Henikoff, S., Henikoff, J. G., Sakai, A., Loeb, G. B., and Ahmad, K. (2009) Genome-wide profiling of salt fractions maps physical properties of chromatin. *Genome Res,* **19**(3), 460–469.

[4] Jin, C., Zang, C., Wei, G., Cui, K., Peng, W., Zhao, K., and Felsenfeld, G. (2009) H3.3/H2A.Z double variant-containing nucleosomes mark 'nucleosome-free regions' of active promoters and other regulatory regions. *Nat Genet,* **41**(8), 941–945.

[5] Svotelis, A., Gévry, N., and Gaudreau, L. (2009) Chromatin immunoprecipitation in mammalian cells. *Methods Mol Biol,* **543**, 243–251.

[6] Zhang, Z. and Pugh, B. F. (2011) High-resolution genome-wide mapping of the primary structure of chromatin. *Cell,* **144**(2), 175–186.

[7] Rodrigue, S., Materna, A. C., Timberlake, S. C., Blackburn, M. C., Malmstrom, R. R., Alm, E. J., and Chisholm, S. W. (2010) Unlocking short read sequencing for metagenomics. *PLoS One,* **5**(7), e11840.

[8] Li, H. and Durbin, R. (2009) Fast and accurate short read alignment with Burrows-Wheeler transform. *Bioinformatics,* **25**(14), 1754–1760.

[9] Li, H., Handsaker, B., Wysoker, A., Fennell, T., Ruan, J., Homer, N., Marth, G., Abecasis, G., Durbin, R., and Subgroup, . G. P. D. P. (2009) The Sequence Alignment/Map format and SAMtools. *Bioinformatics,* **25**(16), 2078–2079.

[10] Zhang, Y., Liu, T., Meyer, C. A., Eeckhoute, J., Johnson, D. S., Bernstein, B. E., Nusbaum, C., Myers, R. M., Brown, M., Li, W., and Liu, X. S. (2008) Model-based analysis of ChIP-Seq (MACS). *Genome Biol,* **9**(9), R137.

[11] Tan, S. K., Lin, Z. H., Chang, C. W., Varang, V., Chng, K. R., Pan, Y. F., Yong, E. L., Sung, W. K., Sung, W. K., and Cheung, E. (2011) AP-2? regulates oestrogen receptor-mediated long-range chromatin interaction and gene transcription. *EMBO J,* **30**(13), 2569–2581.

[12] Magnani, L., Ballantyne, E. B., Zhang, X., and Lupien, M. (2011) PBX1 genomic pioneer function drives ER? signaling underlying progression in breast cancer. *PLoS Genet,* **7**(11), e1002368.

[13] Fullwood, M. J., Liu, M. H., Pan, Y. F., Liu, J., Xu, H., Mohamed, Y. B., Orlov, Y. L., Velkov, S., Ho, A., Mei, P. H., Chew, E. G. Y., Huang, P. Y. H., Welboren, W.-J., Han, Y., Ooi, H. S., Ariyaratne, P. N., Vega, V. B., Luo, Y., Tan, P. Y., Choy, P. Y., Wansa, K. D. S. A., Zhao, B., Lim, K. S., Leow, S. C., Yow, J. S., Joseph, R., Li, H., Desai, K. V., Thomsen, J. S., Lee, Y. K., Karuturi, R. K. M., Herve, T., Bourque, G., Stunnenberg, H. G., Ruan, X., Cacheux-Rataboul, V., Sung, W.-K., Liu, E. T., Wei, C.-L., Cheung, E., and Ruan, Y. (2009) An oestrogen-receptor-alpha-bound human chromatin interactome. *Nature,* **462**(7269), 58–64.

[14] Zwart, W., Theodorou, V., Kok, M., Canisius, S., Linn, S., and Carroll, J. S. (2011) Oestrogen receptor-co-factor-chromatin specificity in the transcriptional regulation of breast cancer. *EMBO J,* **30**(23), 4764–4776.

[15] Lupien, M., Meyer, C. A., Bailey, S. T., Eeckhoute, J., Cook, J., Westerling, T., Zhang, X., Carroll, J. S., Rhodes, D. R., Liu, X. S., and Brown, M. (2010) Growth factor stimulation induces a distinct ER(alpha) cistrome underlying breast cancer endocrine resistance. *Genes Dev,* **24**(19), 2219–2227.

[16] Schmidt, D., Schwalie, P. C., Ross-Innes, C. S., Hurtado, A., Brown, G. D., Carroll, J. S., Flicek, P., and Odom, D. T. (2010) A CTCF-independent role for cohesin in tissue-specific transcription. *Genome Res,* **20**(5), 578–588.

[17] Varley, K. E., Gertz, J., Bowling, K. M., Parker, S. L., Reddy, T. E., Pauli-Behn, F., Cross, M. K., Williams, B. A., Stamatoyannopoulos, J. A., Crawford, G. E., Absher, D. M., Wold, B. J., and Myers, R. M. (2013) Dynamic DNA methylation across diverse human cell lines and tissues. *Genome Res,* **23**(3), 555–567.

[18] Hoffman, M. M., Ernst, J., Wilder, S. P., Kundaje, A., Harris, R. S., Libbrecht, M., Giardine, B., Ellenbogen, P. M., Bilmes, J. A., Birney, E., Hardison, R. C., Dunham, I., Kellis, M., and Noble, W. S. (2013) Integrative annotation of chromatin elements from ENCODE data. *Nucleic Acids Res,* **41**(2), 827–841.

[19] Tropberger, P., Pott, S., Keller, C., Kamieniarz-Gdula, K., Caron, M., Richter, F., Li, G., Mittler, G., Liu, E. T., Bühler, M., Margueron, R., and Schneider, R. (2013) Regulation of transcription through acetylation of H3K122 on the lateral surface of the histone octamer. *Cell,* **152**(4), 859–872.

[20] He, H. H., Meyer, C. A., Chen, M. W., Jordan, V. C., Brown, M., and Liu, X. S. (2012) Differential DNase I hypersensitivity reveals factor-dependent chromatin dynamics. *Genome Res,* **22**(6), 1015–1025.

[21] Li, G., Ruan, X., Auerbach, R. K., Sandhu, K. S., Zheng, M., Wang, P., Poh, H. M., Goh, Y., Lim, J., Zhang, J., Sim, H. S., Peh, S. Q., Mulawadi, F. H., Ong, C. T., Orlov, Y. L., Hong, S., Zhang, Z., Landt, S., Raha, D., Euskirchen, G., Wei, C.-L., Ge, W., Wang, H., Davis, C., Fisher-Aylor, K. I., Mortazavi, A., Gerstein, M., Gingeras, T., Wold, B., Sun, Y., Fullwood, M. J., Cheung, E., Liu, E., Sung, W.-K., Snyder, M., and Ruan, Y. (2012) Extensive promoter-centered chromatin interactions provide a topological basis for transcription regulation. *Cell,* **148**(1-2), 84–98.

[22] Quinlan, A. R. and Hall, I. M. (2010) BEDTools: a flexible suite of utilities for comparing genomic features. *Bioinformatics,* **26**(6), 841–842.

[23] Hah, N., Danko, C. G., Core, L., Waterfall, J. J., Siepel, A., Lis, J. T., and Kraus, W. L. (2011) A rapid, extensive, and transient transcriptional response to estrogen signaling in breast cancer cells. *Cell,* **145**(4), 622–634.

[24] McLean, C. Y., Bristor, D., Hiller, M., Clarke, S. L., Schaar, B. T., Lowe, C. B., Wenger, A. M., and Bejerano, G. (2010) GREAT improves functional interpretation of cis-regulatory regions. *Nat Biotechnol,* **28**(5), 495–501.

[25] Ye, T., Krebs, A. R., Choukrallah, M.-A., Keime, C., Plewniak, F., Davidson, I., and Tora, L. (2011) seqMINER: an integrated ChIP-seq data interpretation platform. *Nucleic Acids Res,* **39**(6), e35.

[26] Barski, A., Cuddapah, S., Cui, K., Roh, T.-Y., Schones, D. E., Wang, Z., Wei, G., Chepelev, I., and Zhao, K. (2007) High-resolution profiling of histone methylations in the human genome. *Cell,* **129**(4), 823–837.

[27] Heintzman, N. D., Stuart, R. K., Hon, G., Fu, Y., Ching, C. W., Hawkins, R. D., Barrera, L. O., Calcar, S. V., Qu, C., Ching, K. A., Wang, W., Weng, Z., Green, R. D., Crawford, G. E., and Ren, B. (2007) Distinct and predictive chromatin signatures of transcriptional promoters and enhancers in the human genome. *Nat Genet,* **39**(3), 311–318.

[28] Ernst, J. and Kellis, M. (2010) Discovery and characterization of chromatin states for systematic annotation of the human genome. *Nat Biotechnol,* **28**(8), 817–825.

[29] Pekowska, A., Benoukraf, T., Zacarias-Cabeza, J., Belhocine, M., Koch, F., Holota, H., Imbert, J., Andrau, J.-C., Ferrier, P., and Spicuglia, S. (2011) H3K4 tri-methylation provides an epigenetic signature of active enhancers. *EMBO J,* **30**(20), 4198–4210.

[30] Hu, G., Cui, K., Northrup, D., Liu, C., Wang, C., Tang, Q., Ge, K., Levens, D., Crane-Robinson, C., and Zhao, K. (2013) H2A.Z facilitates access of active and repressive complexes to chromatin in embryonic stem cell self-renewal and differentiation. *Cell Stem Cell,* **12**(2), 180–192.

[31] Ernst, J., Kheradpour, P., Mikkelsen, T. S., Shoresh, N., Ward, L. D., Epstein, C. B., Zhang, X., Wang, L., Issner, R., Coyne, M., Ku, M., Durham, T., Kellis, M., and Bernstein, B. E. (2011) Mapping and analysis of chromatin state dynamics in nine human cell types. *Nature,* **473**(7345), 43–49.

[32] Hardy, S., Jacques, P.-E., Gévry, N., Forest, A., Fortin, M.-E., Laflamme, L., Gaudreau, L., and Robert, F. (2009) The euchromatic and heterochromatic landscapes are shaped by antagonizing effects of transcription on H2A.Z deposition. *PLoS Genet,* **5**(10), e1000687.

[33] Dineen, D. G., Schröder, M., Higgins, D. G., and Cunningham, P. (2010) Ensemble approach combining multiple methods improves human transcription start site prediction. *BMC Genomics,* **11**, 677.

[34] Thurman, R. E., Rynes, E., Humbert, R., Vierstra, J., Maurano, M. T., Haugen, E., Sheffield, N. C., Stergachis, A. B., Wang, H., Vernot, B., Garg, K., John, S., Sandstrom, R., Bates, D., Boatman, L., Canfield, T. K., Diegel, M., Dunn, D., Ebersol, A. K., Frum, T., Giste, E., Johnson, A. K., Johnson, E. M., Kutyavin, T., Lajoie, B., Lee, B.-K., Lee, K., London, D., Lotakis, D., Neph, S., Neri, F., Nguyen, E. D., Qu, H., Reynolds, A. P., Roach, V., Safi, A., Sanchez, M. E., Sanyal, A., Shafer, A., Simon, J. M., Song, L., Vong, S., Weaver, M., Yan, Y., Zhang, Z., Zhang, Z., Lenhard, B., Tewari, M., Dorschner, M. O., Hansen, R. S., Navas, P. A., Stamatoyannopoulos, G., Iyer, V. R., Lieb, J. D., Sunyaev, S. R., Akey, J. M., Sabo, P. J., Kaul, R., Furey, T. S., Dekker, J., Crawford, G. E., and Stamatoyannopoulos, J. A. (2012) The accessible chromatin landscape of the human genome. *Nature,* **489**(7414), 75–82.

[35] Coulombe, C., Poitras, C., Nordell-Markovits, A., Brunelle, M., Lavoie, M.-A., Robert, F., and Jacques, P.-E. (2014) VAP: a versatile aggregate profiler for efficient genome-wide data representation and discovery. *Nucleic Acids Res,* **dx.doi.org/10.1093/nar/gku302**.

[36] Kundaje, A., Kyriazopoulou-Panagiotopoulou, S., Libbrecht, M., Smith, C. L., Raha, D., Winters, E. E., Johnson, S. M., Snyder, M., Batzoglou, S., and Sidow, A. (2012) Ubiquitous heterogeneity and asymmetry of the chromatin environment at regulatory elements. *Genome Res,* **22**(9), 1735–1747.

[37] Chen, K., Xi, Y., Pan, X., Li, Z., Kaestner, K., Tyler, J., Dent, S., He, X., and Li, W. (2013) DANPOS: dynamic analysis of nucleosome position and occupancy by sequencing. *Genome Res,* **23**(2), 341–351.

[38] Heinz, S., Benner, C., Spann, N., Bertolino, E., Lin, Y. C., Laslo, P., Cheng, J. X., Murre, C., Singh, H., and Glass, C. K. (2010) Simple combinations of lineage-determining transcription factors prime cis-regulatory elements required for macrophage and B cell identities. *Mol Cell,* **38**(4), 576–589.

[39] Tang, Q., Chen, Y., Meyer, C., Geistlinger, T., Lupien, M., Wang, Q., Liu, T., Zhang, Y., Brown, M., and Liu, X. S. (2011) A comprehensive view of nuclear receptor cancer cistromes. *Cancer Res,* **71**(22), 6940–6947.

[40] Robinson, J. T., Thorvaldsdóttir, H., Winckler, W., Guttman, M., Lander, E. S., Getz, G., and Mesirov, J. P. (2011) Integrative genomics viewer. *Nat Biotechnol,* **29**(1), 24–26.

[41] Thorvaldsdóttir, H., Robinson, J. T., and Mesirov, J. P. (2013) Integrative Genomics Viewer (IGV): high-performance genomics data visualization and exploration. *Brief Bioinform,* **14**(2), 178–192.

[42] Bock, C., Tomazou, E. M., Brinkman, A. B., Müller, F., Simmer, F., Gu, H., Jäger, N., Gnirke, A., Stunnenberg, H. G., and Meissner, A. (2010) Quantitative comparison of genome-wide DNA methylation mapping technologies. *Nat Biotechnol,* **28**(10), 1106–1114.

Supplementary Tables

Supplementary Table 1, Enriched Motifs
The top 12 most enriched known motifs (38) of ERα w/ and w/o H2A.Z.

Supplementary Table 2, GO Analysis
Gene ontology analysis (24) of the "high-confidence" ERα w/ and w/o H2A.Z un/low-methylated enhancers not pre-bound by ERα in absence of E2.

Supplementary Table 3, Primers
List of the primers used in qPCR analyses.

Supplementary Table 4, Gene Expression Analysis (significant)

Supplementary Table 5, Gene Expression Analysis (all)

Supplementary Legends

Figure S1. High correlation of MNase ChIP-seq signals between independent biological replicates. To ensure the reproducibility of the MNase ChIP-seq experiments, two independent biological replicates of each ChIP-seq experiment were generated and the correlation between reads were computed genome-wide over 2 Kb bins, then 30,000 regions were randomly selected for the final representation. The resulting Pearson correlation coefficient is shown in the right lower corner of each graph (p-value < 0.0001).

Figure S2. Characterization of the relationship between H2A.Z and H3K4me3 at distal and proximal regions. *(A,B)* K-means clustering of proximal *(A, left panel)* or distal *(B, left panel)* H2A.Z enriched regions, the overlap proportion between each cluster and the UCSC's track of CpG islands (right upper panel), and the probability that a TSS is located in each cluster using the 5 bp resolution probability scores for the hg18 genome from the Profisi Ensemble tool (33) (right lower panel). *(C)* Same analysis as Fig. 1A, but including H3K4me3 signal in the K-means clustering analysis. Six distinct clusters (C1 to C6) are derived (left panel). The overlap proportion between each cluster and the CpG islands (right upper panel). The probability that a TSS is located in each cluster (right lower panel).

Figure S3. A complement to Figure 1. *(A)* The average signal profiles of H3K4me1, H3K27ac, H3K4me3 and H2A.Z in 4-Kb window centered on summits of the different TSS-distal non-promoter ERα-groups from Figure 1A are showing no significant difference of the signal before and after E2-stimulation. *(B)* Same analysis as Fig. 1A, but using ERα-consensus-BS identified in all available ChIP-seq data of ERα in MCF-7 cells analyzed by Tang *et al.* (39). This additional analysis confirms the validity of the chromatin states identified in Fig. 1A, as well as the proportion of ERα-active enhancers where H2A.Z is present. *(C)* The kinetics of H2A.Z enrichment after E2-treatment by ChIP-qPCR. Five loci from each ERα w/ and w/o H2A.Z were studied in two independent biological replicates and the results were combined for the final representation (mean +/- SEM). The primers used in these qPCR analysis are listed in Supplementary Table 3.

Figure S4. Genome browser snapshots of ChIP-seq data showing typical examples of both ERα w/ and w/o H2A.Z. The genomic regions were chosen within the 10% strongest ERα-signal. ERα w/ H2A.Z (A) to (E) (upper panels) or w/o H2A.Z (F) to (J) (lower panels) are centered on ERα summit using IGV (Integrative Genomics Viewer, version 2.0.7 (40, 41).

Figure S5. Characterization of TSS-distal non-promoter ERα-BS. *(A)* The overlap proportion between ERα-groups and the different ERE motifs (39). *(B)* To determine if the MNase ChIP-seq data contained enough reads to reach the saturation level in the detection of enriched regions, we used a sub-sampling approach followed by an evaluation of the genome coverage. This method, even though potentially sensitive to antibody's background, was prefered over the saturation in the number of calling peaks by MACS (10) because of the observed effect of frequent peak merging with increasing number of reads and because the whole signal was used in the majority of our analyses. MNase ChIP-seq data were downsampled using Picard command-line tools (http://picard.sourceforge.net), by sub-sampling total reads from 10 to 100% (in increments of 10%). Then the genome coverage was computed for each downsample file using BEDTools (22). The saturation level was approximated by exponential function using GraphPad Prism software. The percentage of reaching the plateau was calculated with the following "one phase association" equation: $y = y_0 + (plateau - y_0) * (1 - e^{-kx})$; where x is the percentage of total reads; y is the percentage of genome coverage; y_0 is the y value when x is zero (note that y_0 was constrained to 0); *plateau* is the y value for which the increase in the number of reads doesn't increase the percentage of the genome coverage; k is the rate constant (expressed in reciprocal of the x axis units). From this equation, the approximation of the percentage of reaching the *plateau* corresponds to the y value when x equal to 100 divided by the *plateau*. *(C)* The ChIP-seq of H2A.Z in MCF-7 cells was downloaded from (19) and used as an independent validation of the

stronger enrichment of H2A.Z in the ERα w/ H2A.Z group compared with ERα w/o H2A.Z. The H2A.Z normalized signal intensity level was analyzed around summits of each ERα-group. The signal of H2A.Z is significantly higher in ERα w/ H2A.Z (-E2, p-value < 0.0001, Mann-Whitney test), thus confirming our classification of ERα-enhancers. *(D)* The normalized ChIP-seq intensity level of H2A.Zac in MCF-7 cells (19) at each ERα-group. The signal of H2A.Zac is significantly higher in ERα w/ H2A.Z (-E2, p-value $<$ 0.0001, Mann-Whitney test). *(E)* The normalized input control intensity level (19) at each ERα-group.

Figure S6. Comparison between the clusters of shapes isolated with H3K4me1 signal around ERα w/ H2A.Z and their corresponding signals. *(A)* Snapshots of typical examples of loci belonging to the nucleosome positioning shape clusters of H3K4me1 centered on summits of ERα w/ H2A.Z (left panel) and w/o H2A.Z (right panel) performed using IGV (Integrative Genomics Viewer, version 2.0.7, (40, 41)). *(B)* As in Figure 3D, but including the 10 largest clusters as well as the corresponding signals of H3K27ac and the MNase-seq input control.

Figure S7. A complement to Figure 3. *(A)* Dominant H3K4me1 signal shapes (-E2, red curves) in the 300 bp window centered around each summit of ERα w/ H2A.Z and the corresponding signals of H3K4me1 (+E2, blue curves), as well as, H3K4me2 (-/+E2, blue curves). The corresponding signal of H3K4me2 correlates with the shapes isolated with H3K4me1 signal confirming that the asymmetric and heterogeneous patterns isolated with H3K4me1 are not an isolated incident of our MNase ChIP-seq data, but can be reproduced using H3K4me2 published by another research group (20). The correlation between H3K4me2 and H3K4me1 signal at ERα w/ H2A.Z confirms that the centered profiles co-exist with side profiles at ERα-enhancers. *(B)* Dominant H2A.Z signal shapes (-E2, red curves) in the 300 bp window centered around each summit of ERα w/ H2A.Z and their corresponding signals of H2A.Z (+E2, blue curves), as well as, H3K4me1 (-/+E2, blue curves). This analysis confirms that the same typical shapes as those isolated with H3K4me1 signal can be identified with H2A.Z signal, and that both signals correlate.

Figure S8. The correlation between the cluster of shapes isolated with paired-end MNase-sequencing signal and their corresponding histone mark signals or H2A.Z signal. Dominant MNase-seq signal shapes (+E2, red curves) in the 300 bp window centered around each summit of ERα w/ H2A.Z and the corresponding signals of MNase-seq (-E2, blue curves), as well as, H3K4me1, H2A.Z and H3K27ac (-/+E2, blue curves). This analysis confirms that MNase ChIP-seq of H3K4me1, H3K27ac or H2A.Z are effective to study the nucleosome positioning around TF-bound enhancers.

Figure S9. A complement to Figure 4. *(A)* Correlation between normalized DNaseI-signal before and after E2-stimulation at ERα w/ and w/o H2A.Z. The resulting Pearson correlation coefficient is shown in the right lower corner of each graph (p-value < 0.0001). *(B)* Since it has been shown that distal ERα/p300/CBP co-occuring regions are enriched by H3K122ac (19), we therefore examined the potential contribution/association of p300/CBP (14) with ERα w/ and w/o H2A.Z. Surprisingly, ERα w/ and w/o H2A.Z overlap $\sim$40-50% with p300/CBP, thus suggesting that H2A.Z are associated more strongly with H3K122ac than with p300/CBP. The mean +/- SEM of the overlap with the two biological replicates of p300 or CBP are represented.

Figure S10. Controls of the DNA methylation analysis. *(A)* To validate the enrichment of CpG dinucleotides in ERα w/ H2A.Z regions, the CpG content in 600 bp windows was normalized by de GC content of each region using the option "CpG" of the "annotatePeaks" usage of HOMER software (version 3.9, (38)). The CpG/GC content remains significantly higher in ERα w/ than w/o H2A.Z (p-value < 0.0001, Mann-Whitney test). *(B)* Proportion of the overlap between ERα-groups and CpG islands track from UCSC. The CpG content of each group is not contaminated by overlapping CpG islands. *(C)* Distribution of the reported proportion of methylated CpG dinucleotides within 600 bp of ERα-summits for each group (left panel), and derived proportions of unmethylated (defined as a reported methylation level below 25%), par-

tially methylated (between 25% and 75%), and fully methylated (above 75%) CpG dinucleotides (right panel). *(D)*. The number of reads from the original study covering each CpG within each ERα-group was analyzed to discard a potential bias in the coverage. The difference in the CpG coverage between ERα w/ and w/o H2A.Z is non-significant (n.s., Mann-Whitney test). *(E)* The number of MspI sites that are successfully covered by RRBS data within all existing MspI sites in each region of the ERα-groups was analyzed to discard potential bias in the coverage. No difference is observed in the CpG coverage between ERα w/ and w/o H2A.Z. However, the RRBS data sets are known to be strongly biased towards CpG richer regions like promoters (42), the fact that $\sim$ 60% of the potential MspI cleavage sites found in ERα-active enhancers were covered in the available RRBS data set suggest that a more complete coverage like the one obtained with whole-genome bisulfite sequencing approaches could reinforce the relationship between H2A.Z and DNA methylation status at enhancers. *(F)* Proportion of the ERα-BS pre-bound by ERα ("Yes") or not ("No"). ERα w/ and w/o H2A.Z show similar proportion of regions pre-bound by unliganded ERα. *(G)* Normalized ChIP-seq intensity level of ERα (-/+E2) at each ERα-group bound by unliganded ERα in the absence of E2. ERα-level is lower in the absence of E2 than in the presence of E2 at these sites. *(H)* Proportion of overlap between ERα-groups pre-bound by ERα in the absence of E2 and the ERα-BS induced by EGF stimulation (15). Activation of ERα by growth factors could thus partly explain the binding of unliganded ERα in the absence of E2. *(I)* As in panel C, except that the groups were split according to their occupancy status by unliganded ERα in the absence of E2. *(J)* As in panel I (left), except that the groups were split according to their distribution within gene bodies or intergenic regions. This result discards a potential bias in the methylation status level caused by difference in the genomic distribution of ERα w/ and w/o H2A.Z, thus confirming that in the absence of pre-binding of ERα, CpG found in ERα w/ H2A.Z are more maintained in an un/low-methylated state than those found in ERα w/o H2A.Z.

Figure S11. Controls of the transcription at enhancers analysis. *(A-C)* As in Figure 6A, except that only the 40 min time point is shown over all ERα-groups *(A)*, or only at genic ERα-active enhancers *(B)*, or at the subset of ERα-active enhancers associated with specific E2-regulated genes *(C)*. This latter result validates that the subset of ERα resulting from the pairing between ERα-enhancers and a specific E2-regulated TSS behaves as the complete set. *(D)* Average profiles of the normalized H2A.Z signal over the TSS specifically associated with ERα-active enhancers.

Figure S12. H2A.Z helps the recruitment of RNA polymerase II and cohesin complex as well as transcription at active enhancers. *(A-B,D-G)* ChIP-qPCR of RNAPII (8WG16 antibody) *(A)*, RNAPII (RPB2 antibody) *(B)*, H2A.Z *(D)*, ERα *(E)*, H2A *(F)*, or RAD21 *(G)* following a 30 min of E2-treatment and the H2A.Z depletion by shRNA at five loci of both ERα w/ H2A.Z (loci (A) to (E)) and ERα w/o H2A.Z (loci (F) to (J)). The results represent the mean +/- SEM of two independent biological replicates. The coordinates of each locus as well as the sequences of the primers are available in the Supplementary Table 3. *(C)* The shRNA construct against H2A.Z efficiently reduces chromatin-associated H2A.Z protein levels, as shown by western blotting.

Figure S13. Genome browser snapshots showing H2A.Z enrichment at ERα w/ H2A.Z (A) to (E) and its potential TSS/gene target(s). *CHSY1*, *SYT12*, *RHOD*, *DAPK2* and *TMPRSS2* are E2-regulated genes according to GRO-seq data. Snapshots have been performed using IGV (Integrative Genomics Viewer, version 2.0.7 (40, 41).

Figure S14. Genome browser snapshots showing H2A.Z enrichment at potential TSS/gene targets associated with ERα w/o H2A.Z (F) to (J). *CCDC88C*, *NMNAT1*, *FAM174B*, *LCMT1* and *NCOR2* are E2-regulated genes according to GRO-seq data. Snapshots have been performed using IGV (Integrative Genomics Viewer, version 2.0.7 (40, 41).

Figure S15. H2A.Z are associated with RAD21 and chromatin loops. *(A-C)* Distribution of normalized RAD21 signal at intergenic *(A)* or complete set *(B)* of ERα-groups, or at the subset of ERα w/ or w/o H2A.Z associated with specific E2-regulated genes *(C)* in the presence of E2. *(D,E)* The normalized ChIA-PET of RNAPII signal at intergenic ERα active enhancers *(D)* or at the subset of ERα w/ or w/o H2A.Z associated with specific E2-regulated genes *(E)* (p-value, Mann-Whitney test).

Figure S16. Average signal profiles. *(A)* Average signal profiles of H3K4me1, H3K27ac, H3K4me3 and H2A.Z in the 4 Kb window around summits of TSS-distal non-promoter ERα-BS. *(B)* Average signal profiles of H3K4me1 in the 300 bp window around summits of TSS-distal non-promoter ERα-BS, ERα w/ H2A.Z or ERα w/o H2A.Z (associated with Fig. 3D, CAGT output).

Figure S17. Optimization of the MNase digestion. *(A)* Electropherogram analysis of a typical MNase titration experiment showing the size distribution of DNA (bp) after digestion using increasing amount of MNase as a function of the detected abondance. The analysis was performed using the 2100 Bioanalyzer instrument (Agilent Technologies). *(B)* Selection of MNase concentration (results of three independent biological replicates). The proportion of mononucleosome (upper panel) as well as the lenght of mononucleosome DNA fragments (lower panel) were computed. The 150 U of MNase was selected for subsequent MNase ChIP-seq experiments, since it gives 80% of mononucleosomes and an expected mononucleosome lenght of the DNA fragments between 145-150 bp.

Supplementary Figures

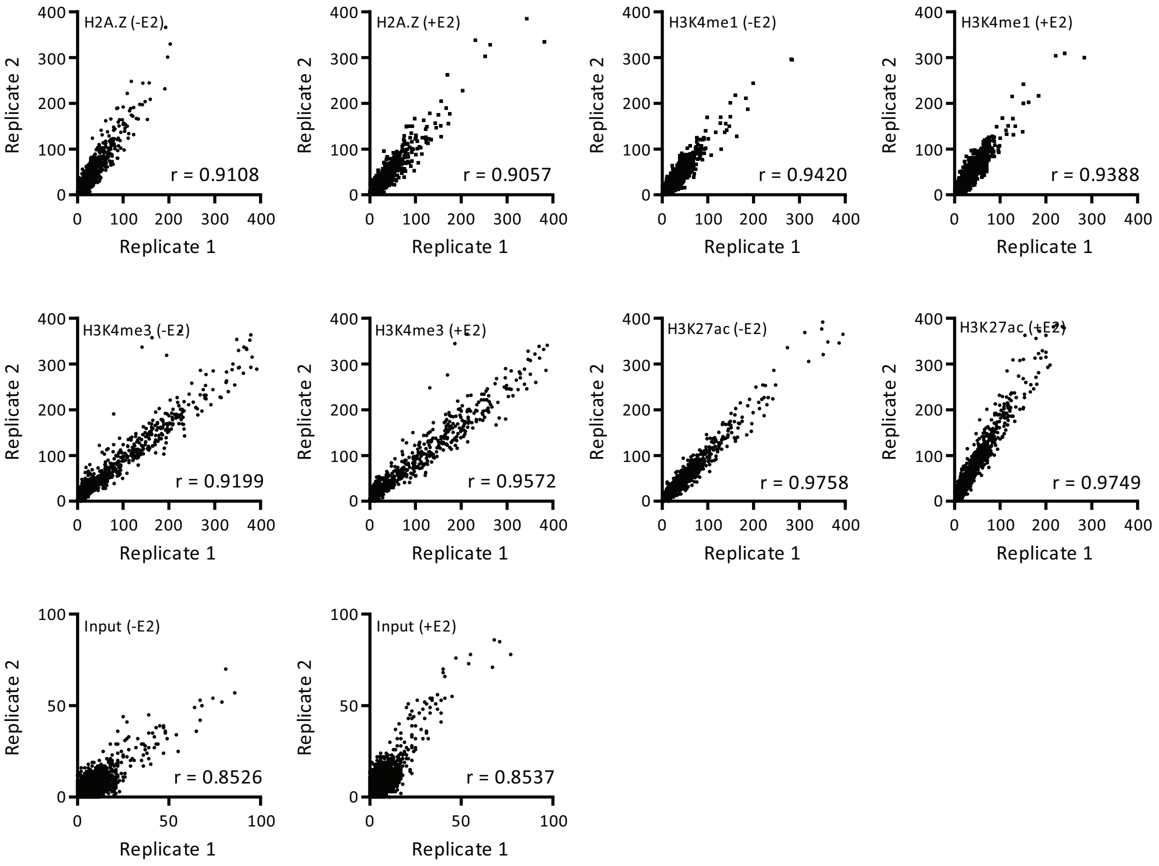

Figure S1: High correlation of MNase ChIP-seq signals between independent biological replicates.

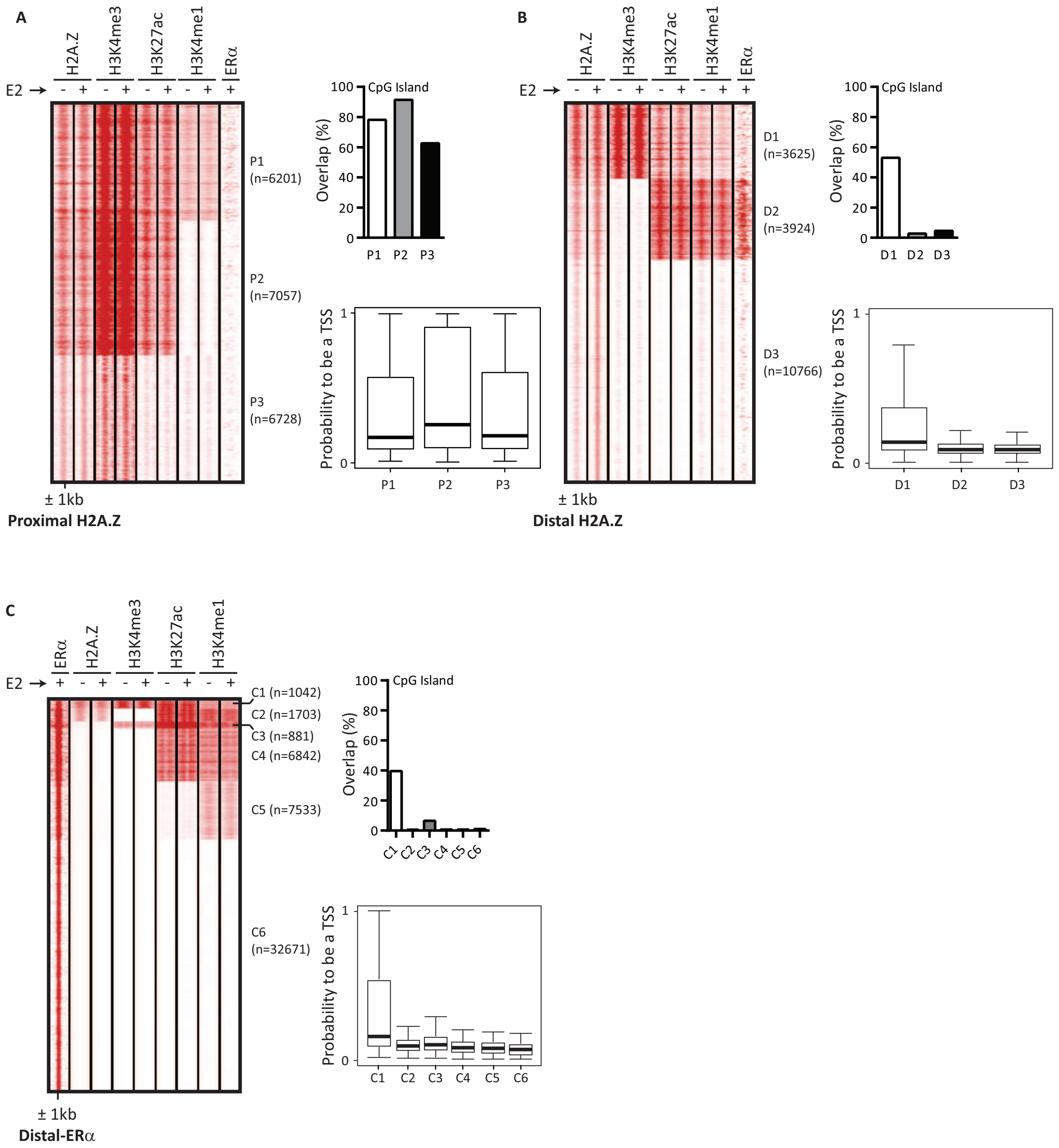

Figure S2: Characterization of the relationship between H2A.Z and H3K4me3 at distal and proximal regions.

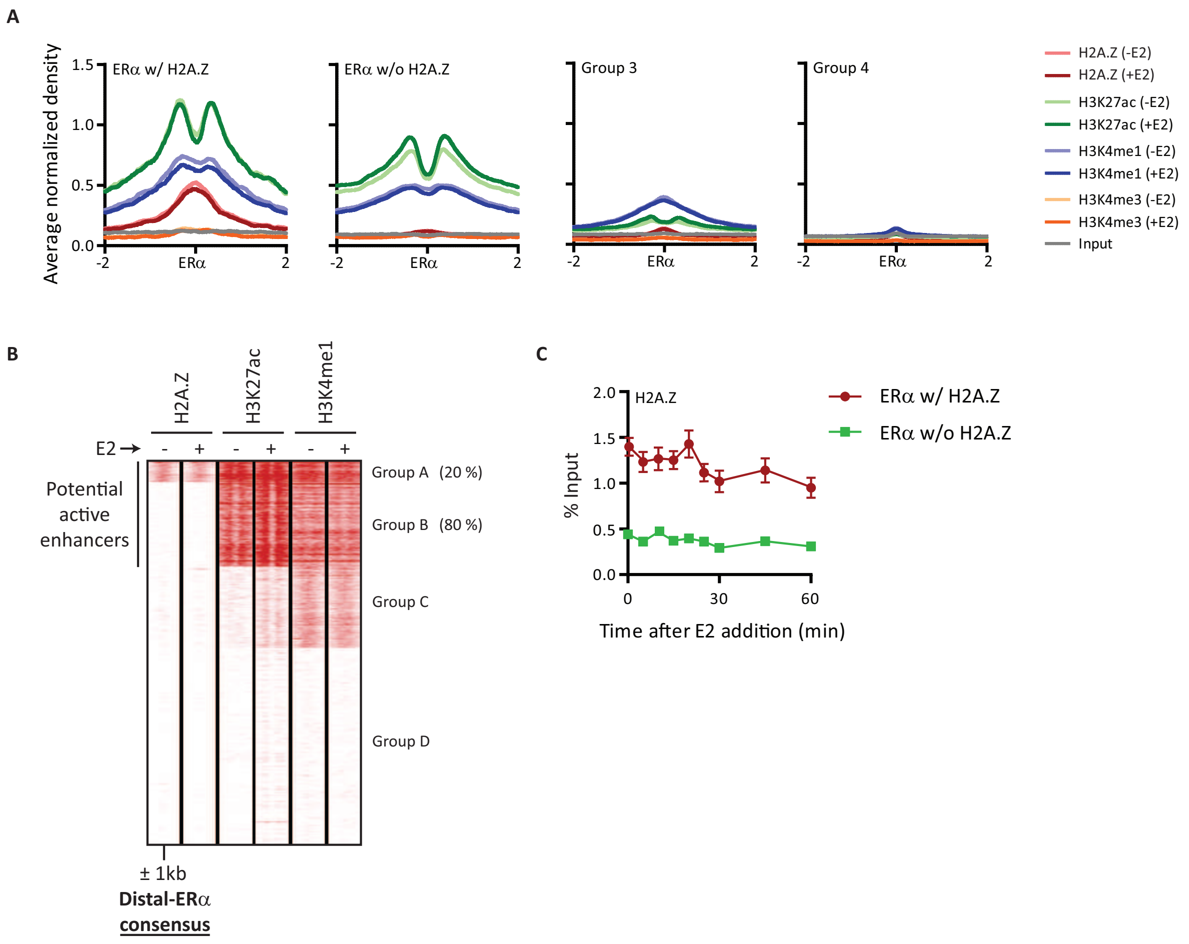

Figure S3: A complement to Figure 1.

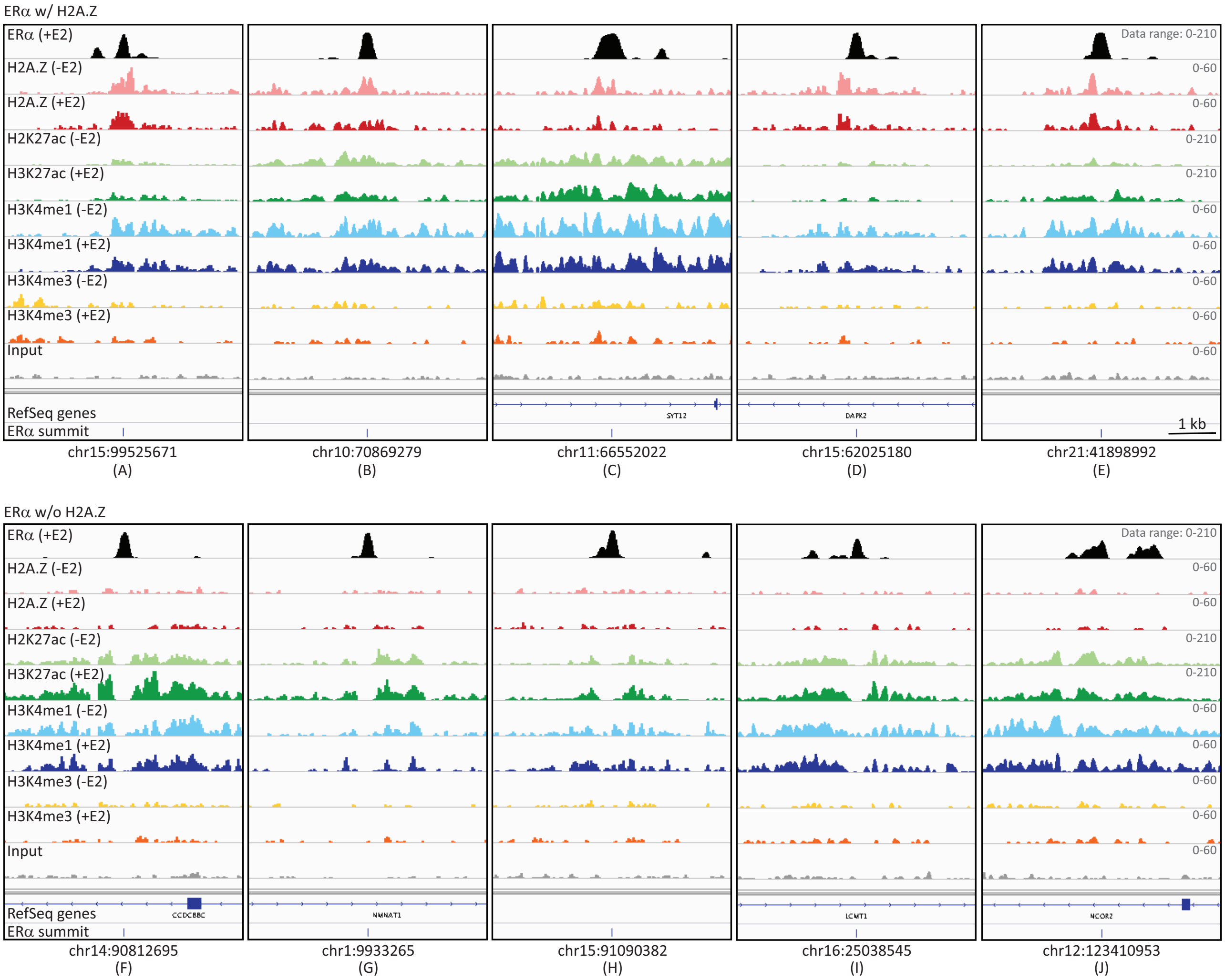

Figure S4: Genome browser snapshots of ChIP-seq data showing typical examples of both ERα w/ and w/o H2A.Z.

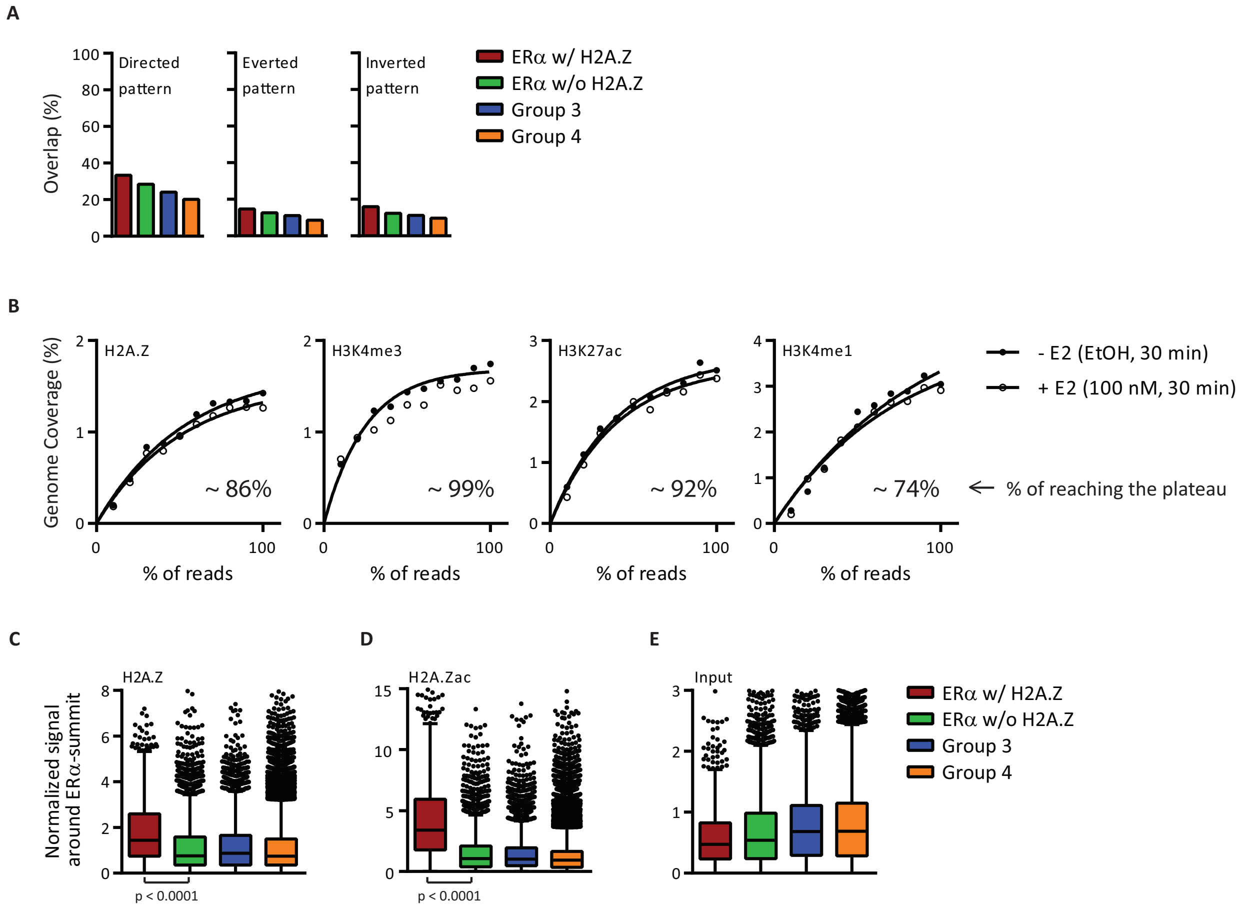

Figure S5: Characterization of TSS-distal non-promoter ERα-BS.

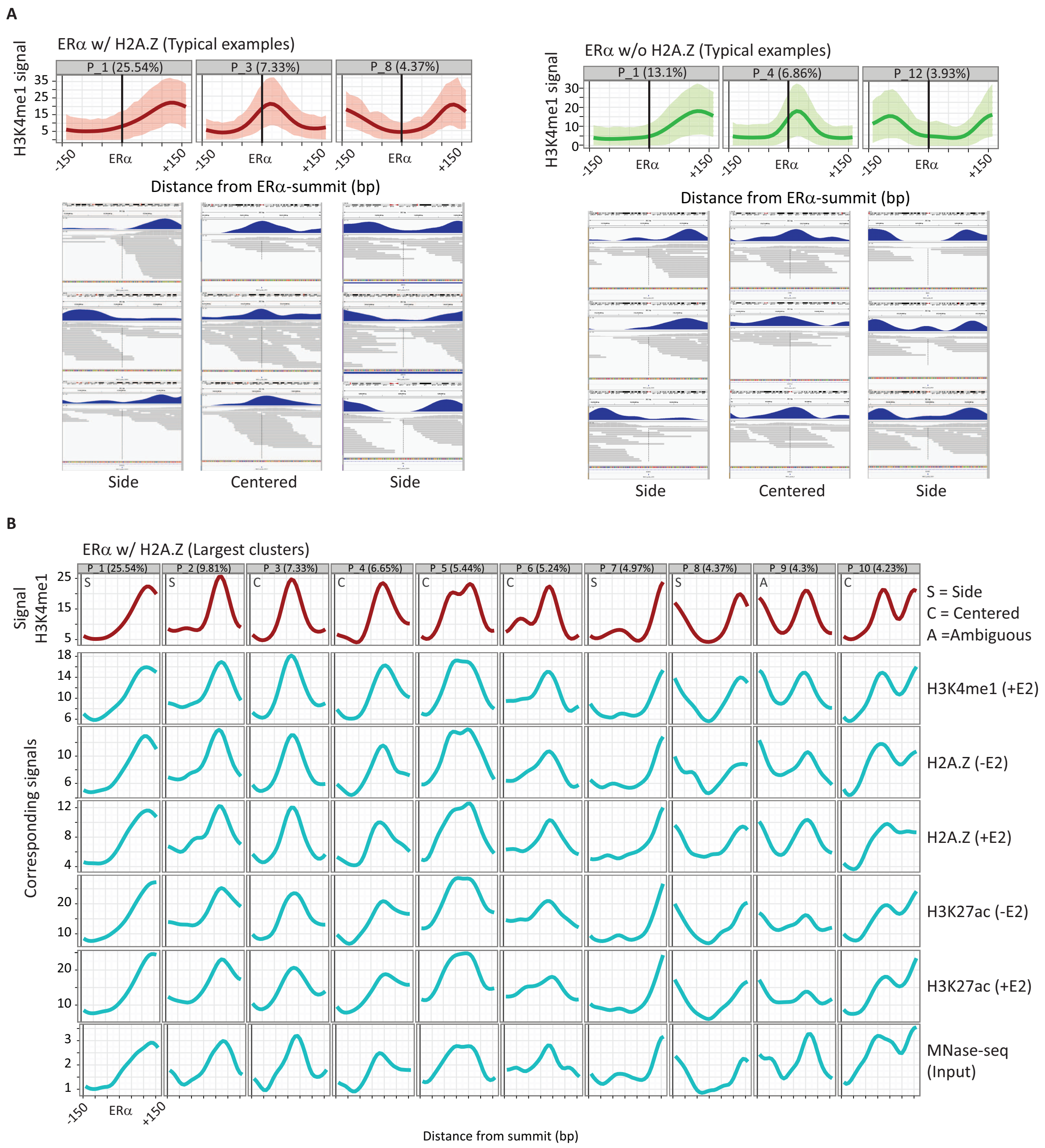

Figure S6: Comparison between the clusters of shapes isolated with H3K4me1 signal around ERα w/ H2A.Z and their corresponding signals.

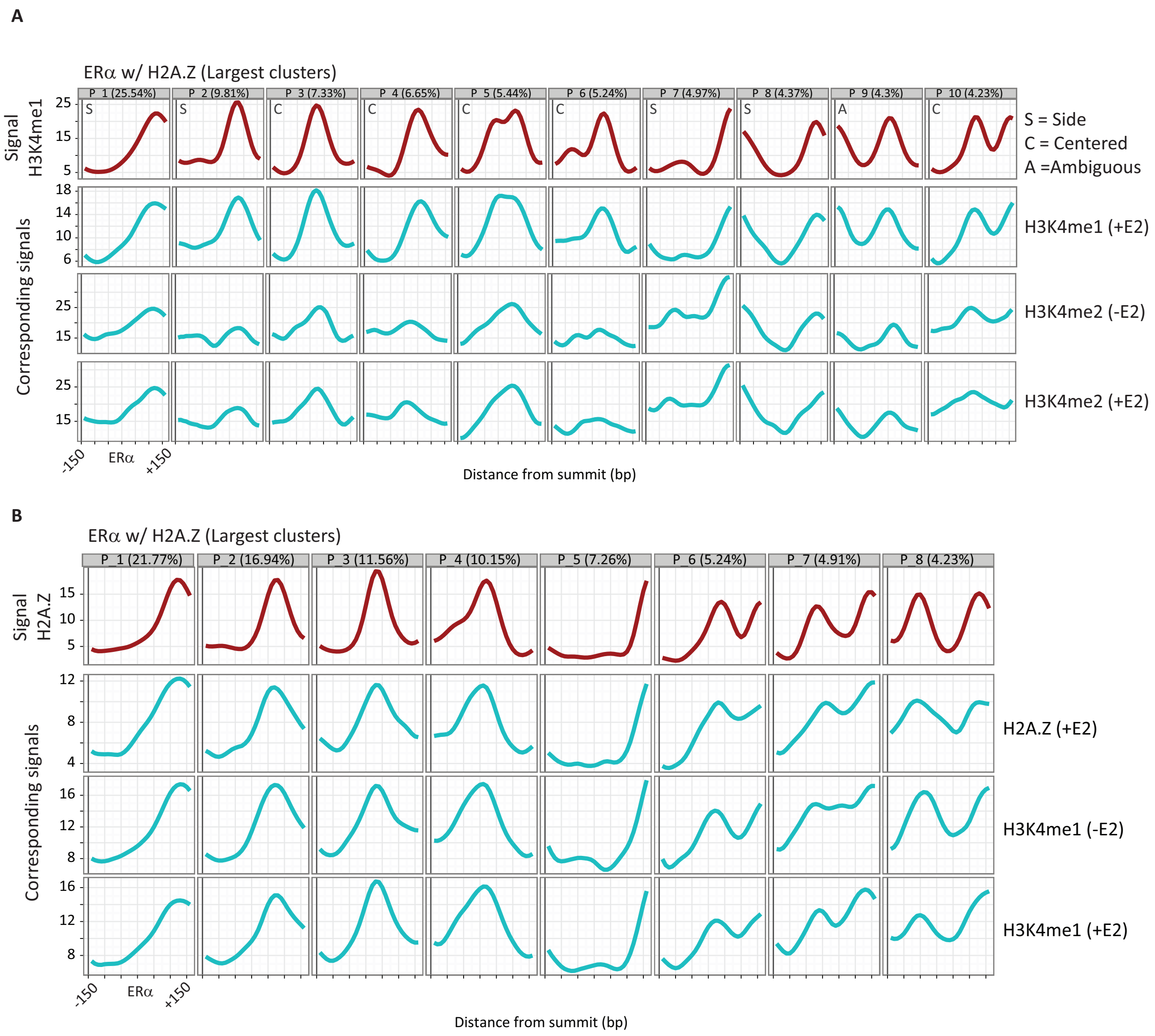

Figure S7: A complement to Figure 3.

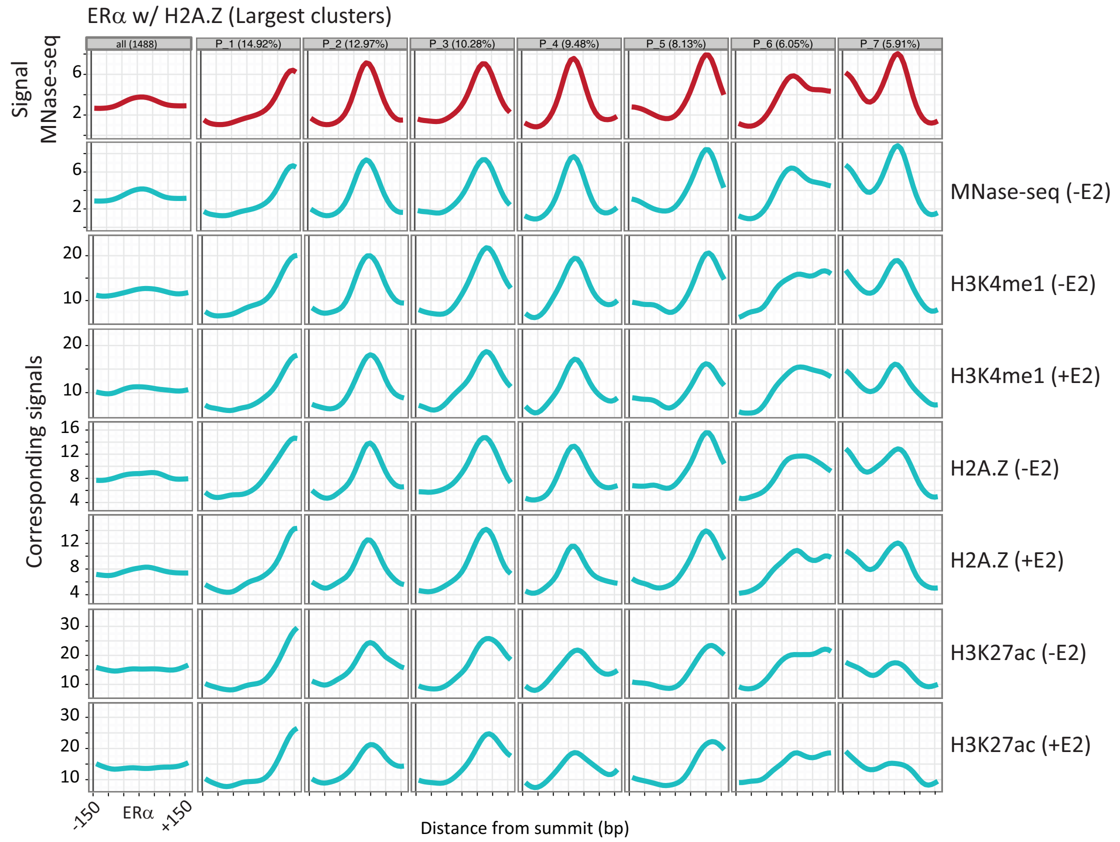

Figure S8: The correlation between the cluster of shapes isolated with paired-end MNase-sequencing signal and their corresponding histone mark signals or H2A.Z signal.

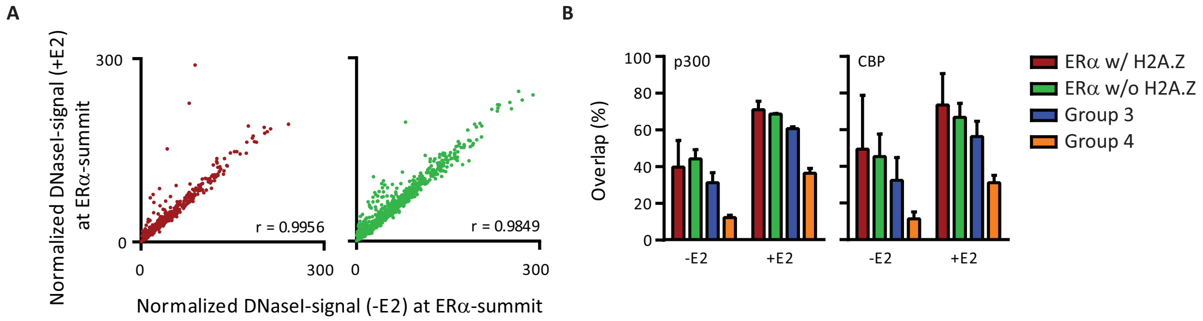

Figure S9: A complement to Figure 4.

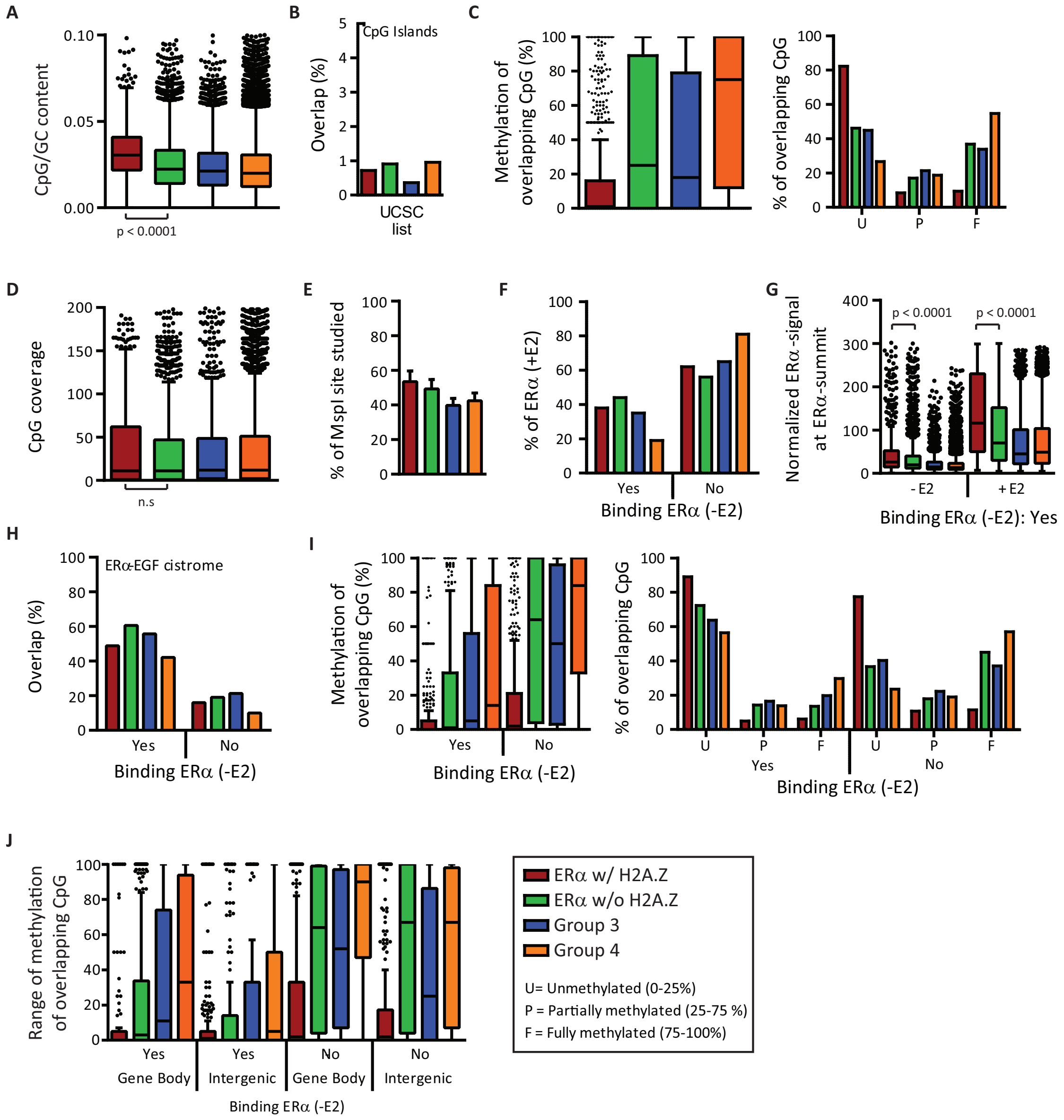

Figure S10: Controls of the DNA methylation analysis.

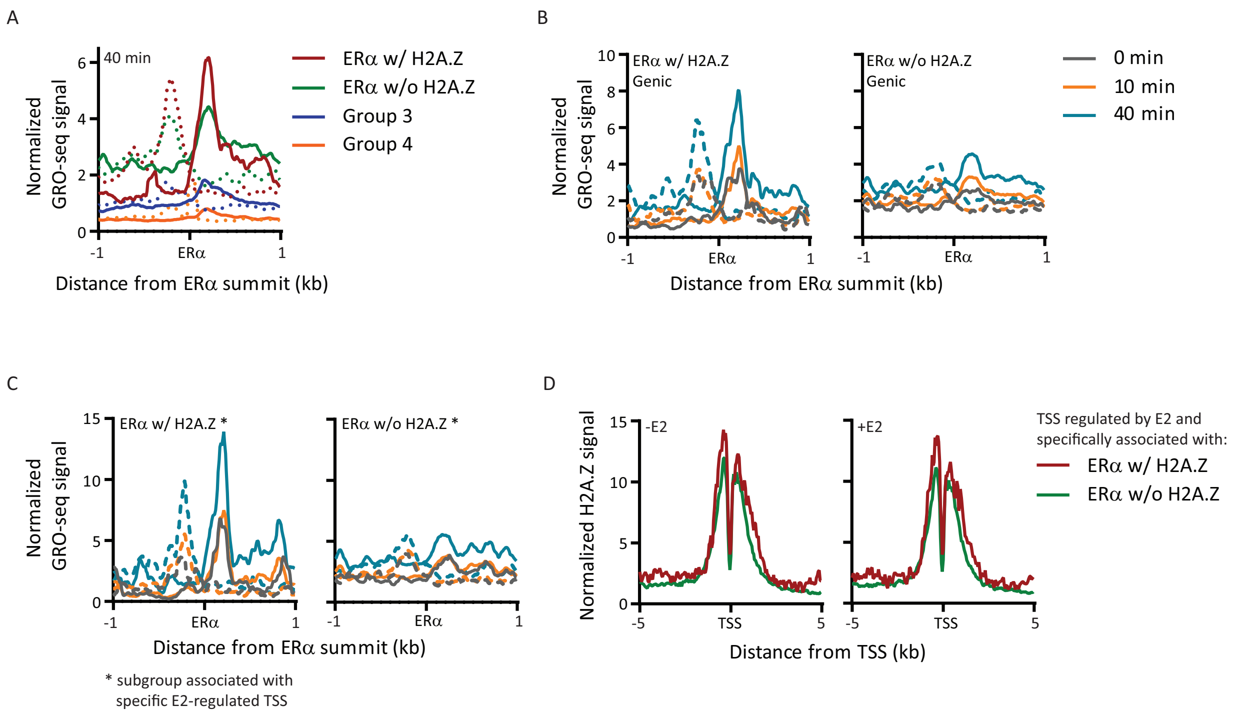

Figure S11: Controls of the transcription at enhancers analysis.

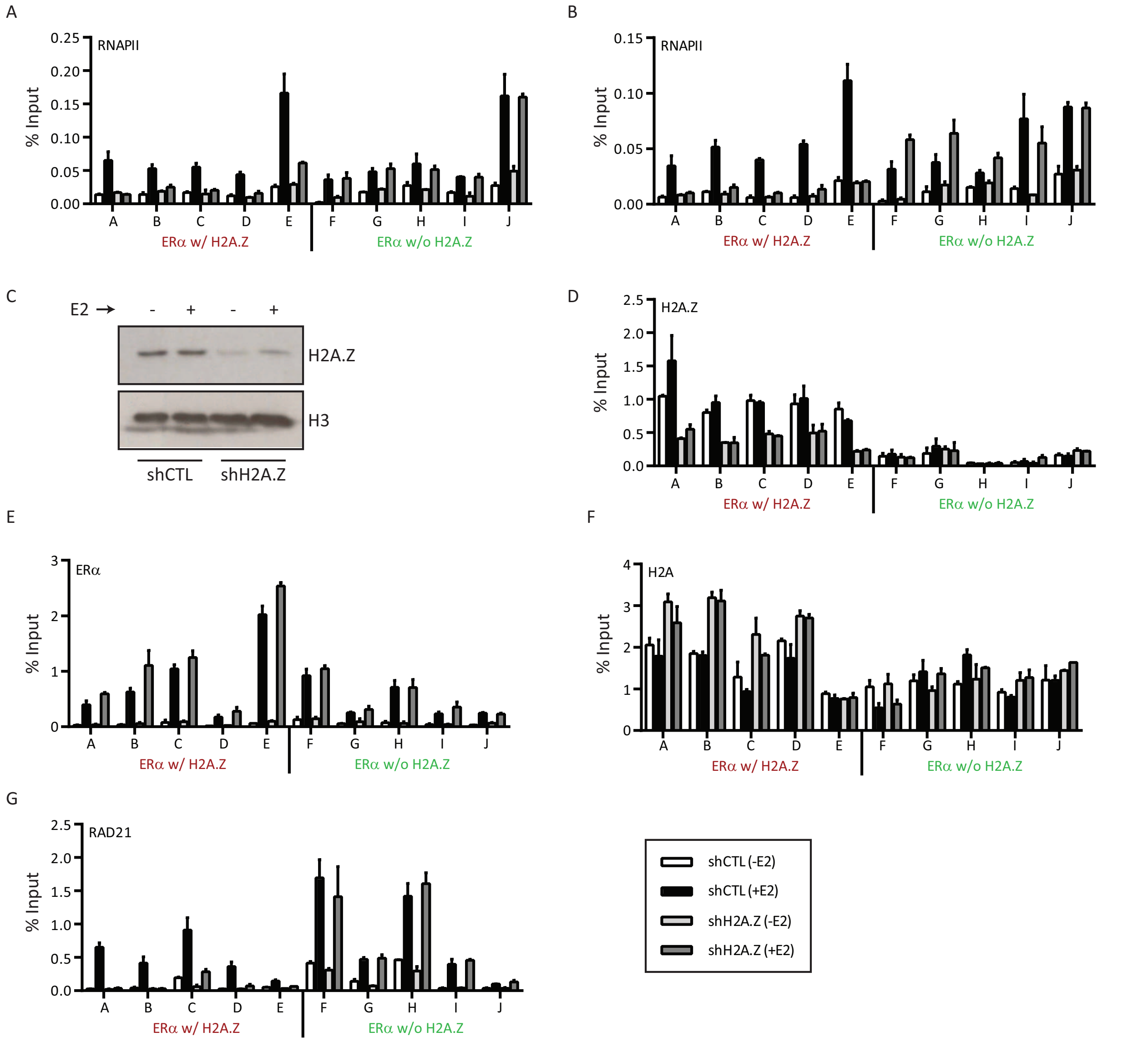

Figure S12: H2A.Z helps the recruitment of RNA polymerase II and cohesin complex as well as transcription at active enhancers.

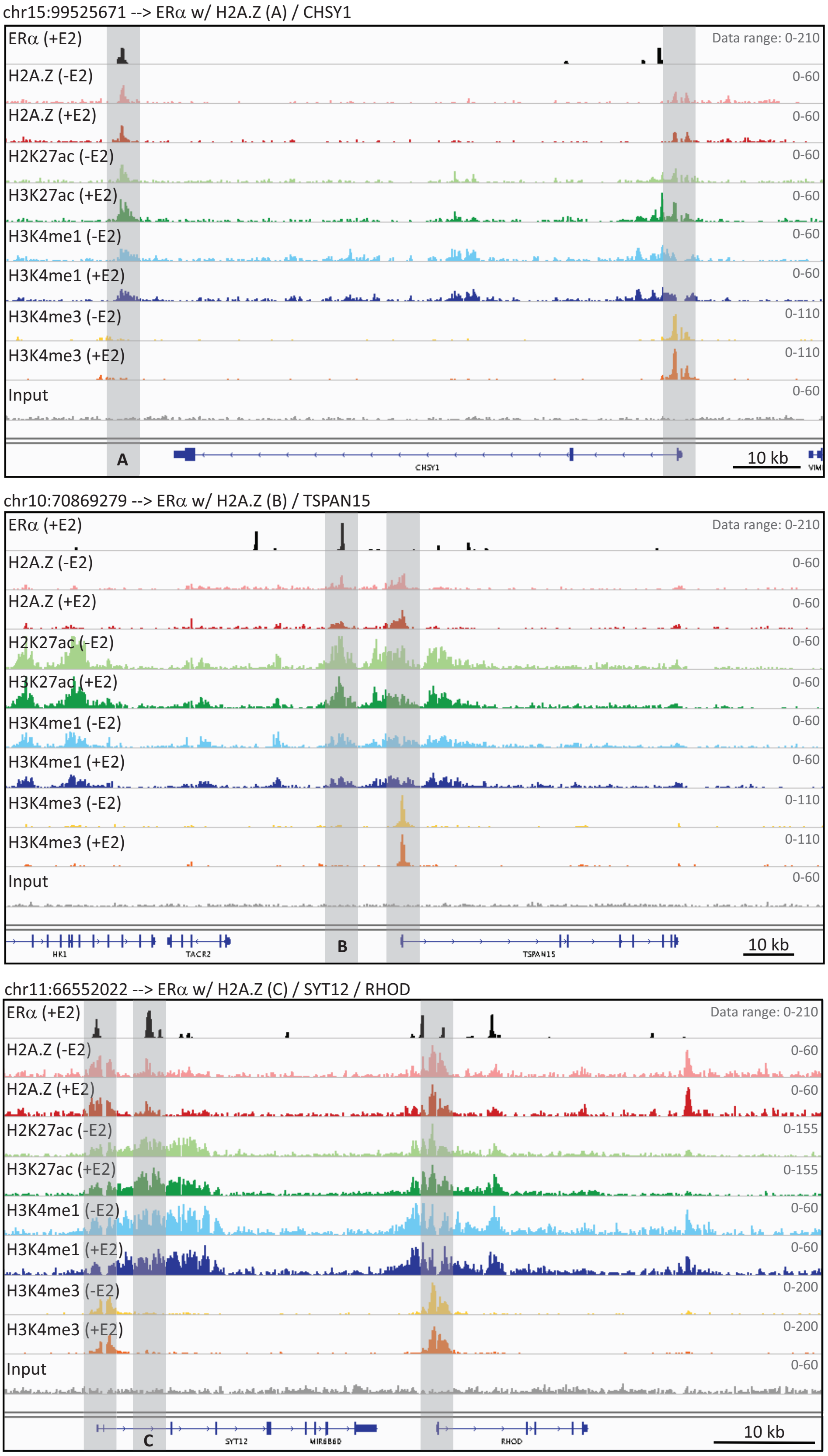
chr15:99525671 --> ERα w/ H2A.Z (A) / CHSY1
ERα (+E2)
Data range: 0-210
H2A.Z (-E2)
0-60
H2A.Z (+E2)
0-60
H2K27ac (-E2)
0-60
H3K27ac (+E2)
0-60
H3K4me1 (-E2)
0-60
H3K4me1 (+E2)
0-60
H3K4me3 (-E2)
0-110
H3K4me3 (+E2)
0-110
Input
0-60
A
CHSY1
10 kb
VIM
chr10:70869279 --> ERα w/ H2A.Z (B) / TSPAN15
ERα (+E2)
Data range: 0-210
H2A.Z (-E2)
0-60
H2A.Z (+E2)
0-60
H2K27ac (-E2)
0-60
H3K27ac (+E2)
0-60
H3K4me1 (-E2)
0-60
H3K4me1 (+E2)
0-60
H3K4me3 (-E2)
0-110
H3K4me3 (+E2)
0-110
Input
0-60
HK1
TACR2
B
TSPAN15
10 kb
chr11:66552022 --> ERα w/ H2A.Z (C) / SYT12 / RHOD
ERα (+E2)
Data range: 0-210
H2A.Z (-E2)
0-60
H2A.Z (+E2)
0-60
H2K27ac (-E2)
0-155
H3K27ac (+E2)
0-155
H3K4me1 (-E2)
0-60
H3K4me1 (+E2)
0-60
H3K4me3 (-E2)
0-200
H3K4me3 (+E2)
0-200
Input
0-60
C
SYT12
MIR6860
RHOD
10 kb

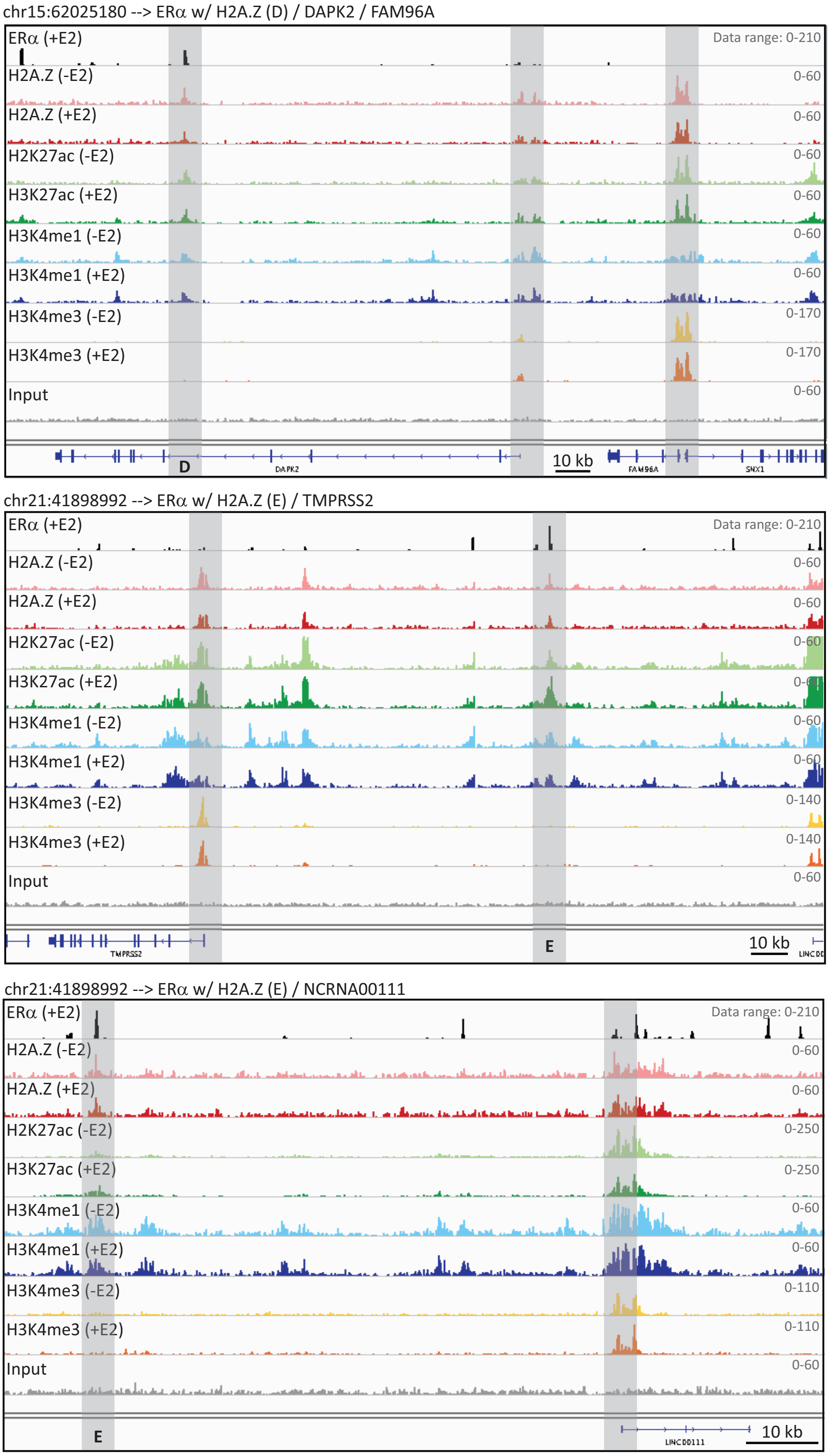

Figure S13: Genome browser snapshots showing H2A.Z enrichment at ERα w/ H2A.Z (A) to (E) and its potential TSS/gene target(s)

chr14:90812695 --> ERα w/o H2A.Z (F) / CCDC88C

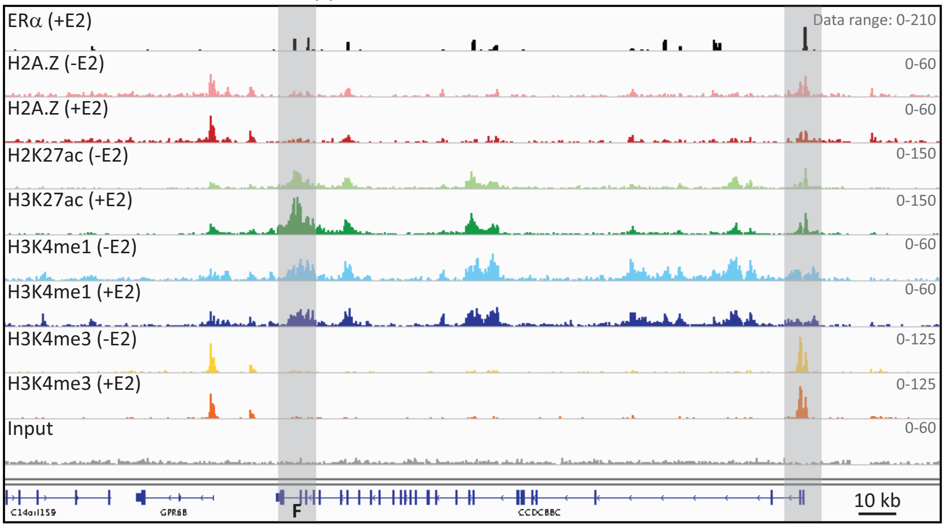

chr1:9933265 --> ERα w/o H2A.Z (G) / NMNAT1

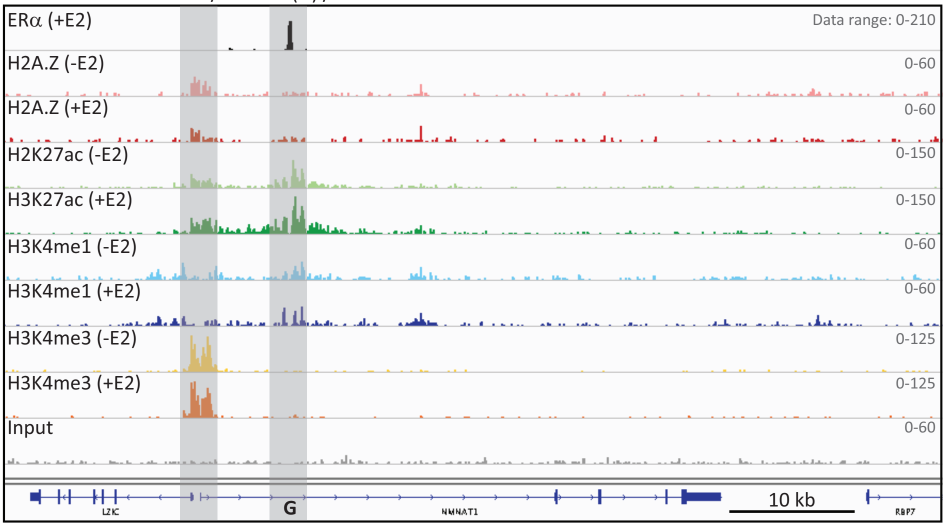

chr15:91090382 --> ERα w/o H2A.Z (H) / FAM174B

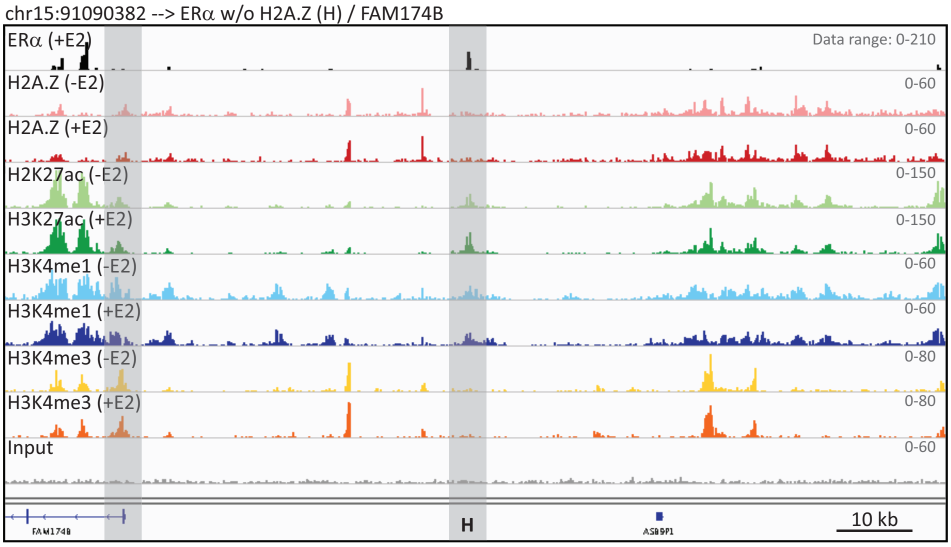

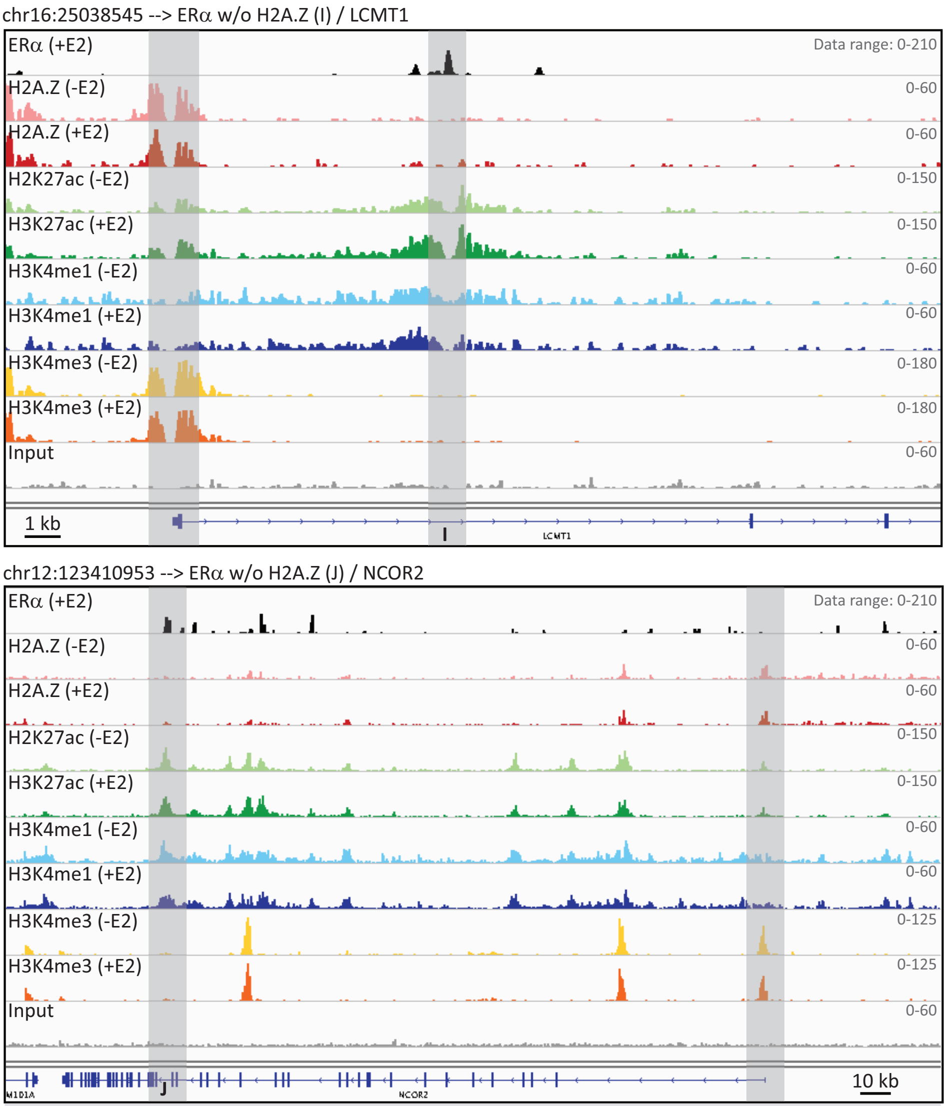

Figure S14: Genome browser snapshots showing H2A.Z enrichment at potential TSS/gene targets associated with ERα w/o H2A.Z (F) to (J)

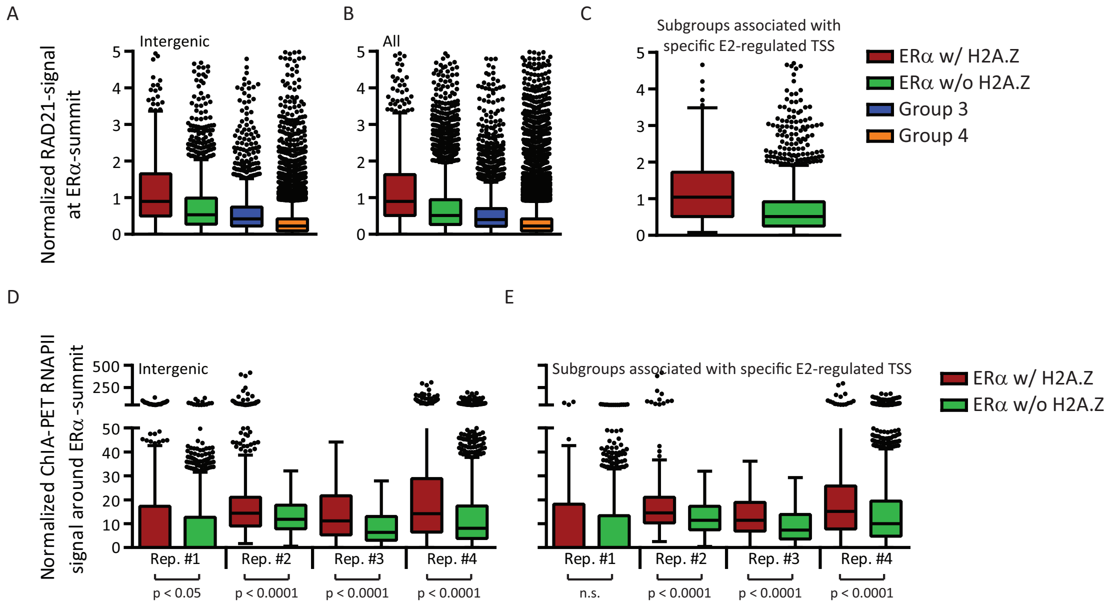

Figure S15: H2A.Z are associated with RAD21 and chromatin loops.

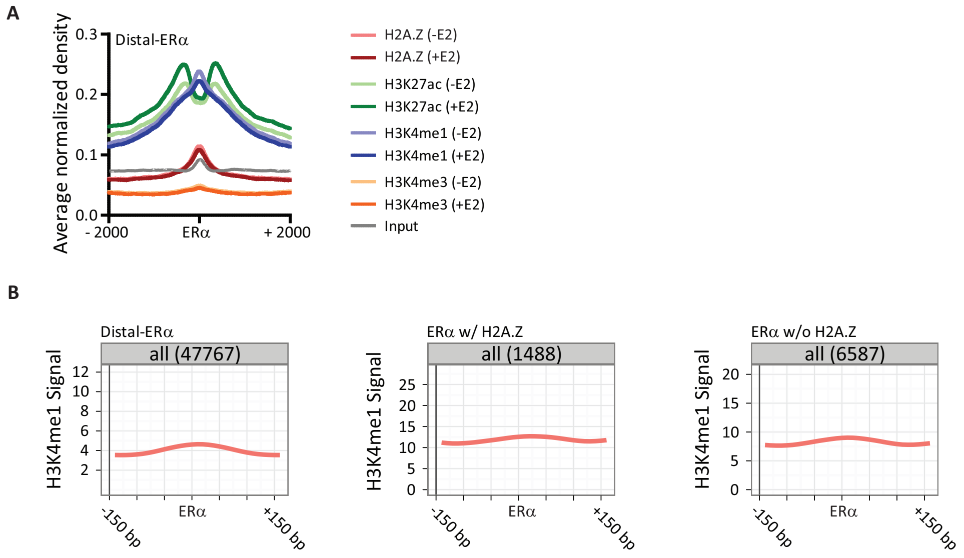

Figure S16: Average signal profiles.

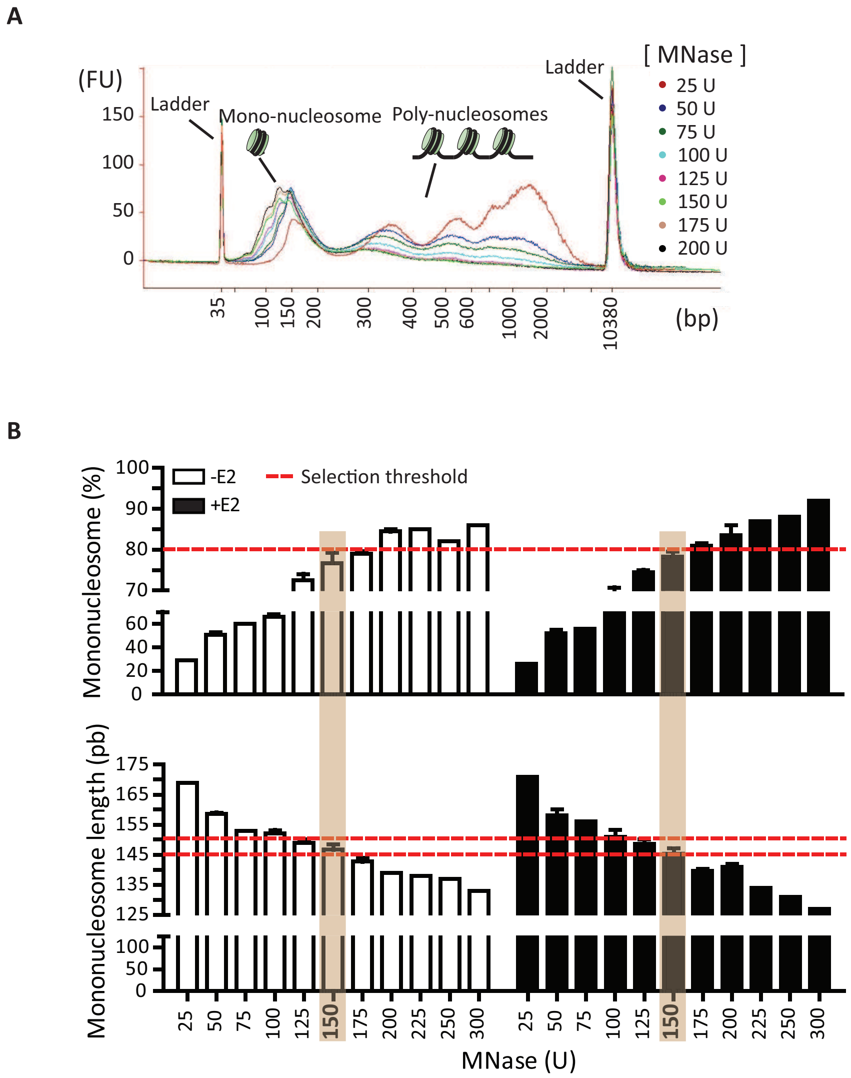

Figure S17: Optimization of the MNase digestion.
